# Parents' Transcultural Attitudes and Knowledge Concerning Human Milk Exchange: A Qualitative Systematic Review and Meta‐Aggregation

**DOI:** 10.1111/mcn.70278

**Published:** 2026-10-27

**Authors:** Julia Will, Vivian Kirchhoff, Julia Lühnen, Jalid Sehouli, Antje Tannen

**Affiliations:** ^1^ Charité – University Medical Center Berlin, corporate member of Freie Universität Berlin and Humboldt Universität zu Berlin Institute of Clinical Nursing Science Berlin Germany; ^2^ Charité – University Medical Center Berlin, corporate member of Freie Universität Berlin and Humboldt Universität zu Berlin Department of Gynecology including center of oncological surgery (CVK) Berlin Germany

**Keywords:** attitude, culture, infant, milk, human, milk banks, religion

## Abstract

If mother's own milk (MOM) is unavailable or insufficient, human milk exchange (HME) on a formal, informal, or commodified basis may be considered. Religion and culture are critical to human milk banking opinions. The review aims to understand parents' transcultural knowledge and attitudes towards HME. The Joanna Briggs Institute (JBI) meta‐aggregation approach was used to synthesize the qualitive evidence. The comprehensive search strategy encompassed systematic research within four databases, hand searching, free web searching, citation searching, and contacting authors. Identified papers were evaluated with the JBI Critical Appraisal Checklist for Qualitative Research. Confidence in the synthesized findings was rated using ConQual. Twenty‐four primary studies were included. These concerned formal, informal, mixed, and unknown HME types. In total, 162 findings were summarized into 16 categories and four synthesized findings. All synthesized findings achieved a moderate ConQual score. HME attitudes and knowledge are informed by religious beliefs, especially milk kinship, and cultural needs, particularly myths. Cultural and religious preferences regarding dyad formation between donor and recipient demonstrate how attitudes and knowledge shape HME acceptance. The identified networks constitute the social context in which attitudes and knowledge develop. Various religious traditions and cultural needs which shape attitudes and knowledge about HME were identified. Heterogeneity within social groups should be considered. Researchers should investigate migration, gender dynamics, and myths. Healthcare professionals (HCP) should consider misconceptions, cultural and religious needs, and social circles. Policymakers should consider developing types of human milk bank (HMB) which are religion‐ and culture‐sensitive.

## Key Messages

- Religion and culture are contributing factors to attitudes and knowledge concerning HME.
- Heterogeneity within cultural and religious groups should be taken into account.
- Researchers should focus on acculturation, migration generations, gender dynamics, and examination of myths.
- Appropriately trained HCPs should correct misconceptions, consider cultural and religious needs, and involve social circles such as religious leaders to reduce uncertainty.
- Policymakers should consider exploring the degree to which cultural and religious traditions are compatible with domestic guidelines, and integrate appropriate adaptations.

**Abbreviations**
DHM: donor human milk
HCP: healthcare professional
HMB: human milk bank
HME: human milk exchange
JBI: Joanna Briggs Institute
MOM: mother's own milk
WHO: World Health Organization

## 1 Introduction

The World Health Organization (WHO) recommends infants are exclusively breastfed up to the age of 6 months (World Health Organization 2025). The skin‐to‐skin contact and emotional bonding that take place during breastfeeding improve mother‐child dyadic interaction (Hass et al. 2025); additionally, mother's own milk (MOM), also called breastmilk, contains nutrients and energy which make it optimal for facilitating health and survival (World Health Organization 2025). The term human milk includes both MOM and donor human milk (DHM), which is expressed breastmilk from a donor (Parker et al. 2026; World Health Organization 2023).

DHM from a human milk bank (HMB) is primarily recommended for infants with (very) low birth weight, in cases where MOM is limited or insufficient (World Health Organization 2023). DHM also improves prognoses in this situation, particularly in reducing cases of necrotizing enterocolitis and bronchopulmonary dysplasia (Huang et al. 2019; Quigley et al. 2024).

Human milk exchange (HME) takes place in various forms: Human milk banking, human milk sharing, and commodified human milk (Palmquist et al. 2019). More than 750 HMBs offering DHM exist (Frauenmilchbank‐Initiative e.V 2025); mothers can voluntarily donate breast milk to these, and then the HMBs collect, test, store, process, and distribute DHM, predominantly to preterm infants (Weaver et al. 2019). Informal milk sharing refers to peer‐to‐peer exchange of human milk via networks such as online platforms (Palmquist et al. 2019), while commodified human milk is traded by individuals or commercial organizations (Palmquist et al. 2019). Private trading is not recommended due to the health risks involved (Food and Drug Administration 2026).

Most HMBs are in North America, Europe, and Brazil, with fewer in South Asia (Israel‐Ballard et al. 2024). Religious and cultural values were identified as important determinants for acceptance of human milk banking (Mathias et al. 2023), with several Muslim mothers in particular indicating increased positivity if their religious needs were considered (Karadag et al. 2015). The number of individuals displaced by war and persecution is at an all‐time high (United Nations High Commissioner for Refugees 2024), and the significance of cultural needs is growing alongside this movement. Empirical findings suggest breastfeeding behavior is affected by a mother's degree of acculturation (Brenne et al. 2018)—where acculturation describes immigrants adopting elements of a different culture (Redfield et al. 1936)—the process of integration and the retention of previous identities are also relevant to this review.

The review takes a global perspective, focusing on parents with diverse transcultural backgrounds. Transculturality arises when individuals with diverse geographic backgrounds interact, where these interactions include dynamic negotiated and transformative processes of behavior and practices (Hock 2012). As Wolfgang Welsch defines them, cultures are heterogeneous groups which influence each other (Hock 2024; Welsch 1992). The review also interprets religions as dynamic instances of positioning which is shaped on culture (Hall 1994; Hock 2012).

The review addresses the research question: What are the attitudes and knowledge levels of parents with different cultural and religious background towards human milk exchange focusing any setting? A comprehensive search strategy was conducted. Primary studies where synthesized by means of a meta‐aggregation using Joanna Briggs Institute (JBI) methodology (Aromataris et al. 2024).

Building on similar reviews (Li et al. 2024; Mathias et al. 2023), this review is necessary in light of the transcultural focus on and diversity‐sensitive recommendations for enhancing equal opportunities in healthcare. Various theoretical frameworks regarding acceptability highlight the relevance of ethnicity and social norms (Ajzen 1991; Sekhon et al. 2017), and as such different types of HME have been considered. Moreover, this meta‐aggregation is distinct in its methodological thoroughness, due to the ConQual score in particular (Aromataris et al. 2024). This review also includes relevant recent studies.

## 2 Methods

The systematic review followed guidance from the JBI (Aromataris et al. 2024) and the Cochrane Handbook for Systematic Reviews of Interventions (Higgins et al. 2024). The manuscript follows the ENTREQ reporting guideline (Enhancing transparency in reporting the synthesis of qualitative research) (Tong et al. 2012). The procedure was based on an *a priori* protocol which is registered with PROSPERO (CRD42024564218). Regular team discussions and journaling were conducted for the purpose of reflexivity (Aromataris et al. 2024).

The comprehensive sensitive search strategy was pre‐planned with the help of experienced research information specialists (Supporting file 1) (Aromataris et al. 2024; Lockwood et al. 2015) and reviewed by one researcher using the PRESS standard (McGowan et al. 2016). Four databases (PubMed, CINAHL via EBSCOhost, Embase via Ovid, Medline via Ovid) were screened for keywords and database‐specific terms. Initial searching of these databases was conducted in January 2024, with no restrictions on language, date range, and study design at that point. The search strategy was discussed in collaboration with six experts in diversity and gynecology for the purpose of establishing which academic journals were relevant; these experts were identified during prior research. Four culture‐specific journals were hand searched: *Zeitschrift für Medizin, Ethik & Islam, Zeitschrift für Medizinische Ethik, Journal of Religion and Health*, and *Journal of Transcultural Nursing*. The research was extended to gray literature, registries, preprint servers, and internet resources such as Google Scholar via Harzing, ResearchGate, Bielefeld Academic Search Engine, medRxiv, Deutsches Register Klinischer Studien, and clinicaltrials.gov. The data were collected and de‐duplicated via EndNote.

The screening process applied inclusion and exclusion criteria (Table 1). Searches were restricted to primary studies written in English. To enable examination of historical patterns, there was no limitation on publication date. Rayyan 2020 was used (Ouzzani et al. 2016). For title and abstract screening, two reviewers screened independently, with any disagreements resolved by discussion. Full‐text screening began with a pilot in which 10% of the studies were independently screened by two reviewers. In line with the AMSTAR 2 checklist, on the basis of an agreement rate exceeding 80%, the remaining studies were screened by a single reviewer (Shea et al. 2017). Exclusions were verified by a second reviewer, and uncertainties were resolved by discussion. Furthermore, forward and backward citation searching was conducted using the Web of Science Core Collection and CitationChaser (Haddaway et al. 2021). The entire literature search was completed in June 2024. In March 2026, a search update in the four databases was carried out by two independent researchers.

**Table 1:** Inclusion and exclusion criteria (Aromataris et al. 2024).

Criteria	Inclusion criteria	Exclusion criteria
Population	(Expectant) parents until 2 years postpartum	Ethnological or religious experts, healthcare professionals
Phenomena of interest	Religion‐related and culture‐related attitudes and knowledge towards formal and informal milk HME	
Context	Clinical and non‐clinical settings	
Type of studies	Qualitative studies and mixed methods studies (ethnography, phenomenology, qualitative inquiry, action research, discourse analysis, and grounded theory)	
Others		Focus on illnesses (Covid‐19, HIV) or palliative context

Qualitative and mixed‐design primary studies were checked using the JBI Critical Appraisal Checklist for Qualitative Research (Aromataris et al. 2024; Lockwood et al. 2015), though in mixed studies only qualitative data was examined. This tool seems to be the most coherent due to its focus on congruity (Hannes et al. 2010). Three reviewers applied the tool independently, judging each study on the basis of ten different items (Table 2). Disagreements were resolved by discussion (Aromataris et al. 2024; Lockwood et al. 2015).

MAXQDA was used to extract the data (VERBI Software 2024). Relevant information was coded line by line by two independent reviewers, with disagreements resolved by discussion. General details were extracted: Data were coded and assigned to categories based on the items in the standardized JBI data extraction tool. All data relevant to the research question were coded by means of repeated reading (Aromataris et al. 2024).

**Table 2:** Quality appraisal (Aromataris et al. 2024).

Study	Q1	Q2	Q3	Q4	Q5	Q6	Q7	Q8	Q9	Q10	Total score
Ahmed et al. (2024)	N/A	Y	Y	Y	Y	Y	Y	Y	Y	Y	9
Herdy Alves et al. (2013)	Y	Y	Y	Y	Y	N	N	Y	Y	Y	8
Bressler et al. (2020)	Y	Y	Y	Y	Y	Y	N	Y	Y	Y	9
Chugh Sachdeva et al. (2019)	U	Y	Y	Y	Y	N	Y	Y	Y	Y	8
Coutsoudis et al. (2011)	U	Y	Y	Y	Y	N	Y	Y	Y	Y	8
Esquerra‐Zwiers et al. (2016)	U	Y	Y	Y	Y	N	Y	Y	N	Y	7
Faruqui et al. (2023)	U	Y	Y	Y	Y	N	Y	Y	Y	Y	8
Gelano et al. (2018)	U	Y	Y	Y	Y	N	Y	Y	Y	Y	8
Gribble (2013)	Y	Y	Y	Y	Y	N	Y	Y	Y	Y	9
Gribble (2014)	U	Y	Y	Y	Y	N	N	Y	Y	Y	7
Karacan et al. (2024)	U	Y	Y	Y	Y	N	Y	Y	Y	Y	8
Karami et al. (2025)	U	Y	Y	Y	Y	Y	Y	Y	Y	Y	9
Kürtüncü et al. (2024)	Y	Y	Y	Y	Y	N	N	Y	Y	Y	8
Loh et al. (2023)	U	Y	Y	Y	Y	N	Y	Y	Y	Y	8
Lubbe et al. (2019)	U	Y	Y	Y	Y	N	N	Y	Y	Y	7
Mackenzie et al. (2013)	U	Y	Y	Y	Y	N	Y	Y	Y	Y	8
Magowan et al. (2020)	U	Y	Y	Y	Y	N	Y	Y	Y	Y	8
Mampane and Wolvaardt (2024)	Y	Y	Y	Y	Y	N	Y	Y	Y	Y	9
Mondkar et al. (2018)	U	Y	Y	Y	Y	N	Y	Y	Y	N	7
O´Sullivan et al. (2016)	U	Y	Y	Y	Y	N	Y	Y	N	Y	8
Oreg and Negev (2023)	U	Y	Y	Y	Y	N	Y	Y	N	N	6
Oueidat et al. (2020)	U	Y	Y	Y	Y	N	Y	Y	N	Y	7
Rabinowitz et al. (2018)	U	Y	Y	Y	Y	N	N	Y	Y	Y	7
Vesel et al. (2023)	U	Y	Y	Y	Y	Y	Y	Y	Y	Y	9

Q1: Congruity between the stated philosophical perspective and the research methodology; Q2: Congruity between the research methodology and the research question or objectives; Q3: Congruity between the research methodology and the methods used to collect data; Q4: Congruity between the research methodology and the representation and analysis of data; Q5: There is congruence between the research methodology and the interpretation of results; Q6: Locating the researcher culturally or theoretically; Q7: Influence of the researcher on the research, and vice‐versa, is addressed; Q8: Representation of participants and their voices; Q9: Ethical approval by an appropriate body; Q10: Relationship of conclusions to analysis, or interpretation of the data; Y‐Yes, N‐No, U‐Unclear, N/A‐Not Applicable.

One researcher classified the data extracts which answer the research question as *finding* for verbatim information from the author or *illustration* for verbatim statements of participants' opinions. Findings were examined to ascertain if they were accompanied by an illustration. Each finding was evaluated for credibility and categorized into unequivocal, credible or not supported. Unequivocal and credible findings were included in the synthesis (Aromataris et al. 2024). The assignments were checked by two independent researchers. Disagreements were resolved by discussion.

The data synthesis was initially completed by one reviewer. The findings were first summarized inductively into categories, each with at least two findings. These categories were then summarized into synthesized findings with at least two categories. A description was developed for each category and synthesized finding (Aromataris et al. 2024). The results were checked by independent reviewers. Disagreements were resolved by discussion.

ConQual was applied to rate the confidence in the synthesized qualitative research (Aromataris et al. 2024; Munn et al. 2014). Dependability of each synthesized finding was checked: The studies were classified by means of selected critical appraisal questions including congruity between the methodology and the research question or objectives, congruity between the methodology and the methods used to collect data, congruity between the methodology and the representation and analysis of data, locating the researcher culturally or theoretically and influence of the researcher, and vice‐versa. The synthesized findings were then downgraded based on the dependability of the included studies. Credibility of the synthesized findings was assessed by rating the analogousness of authors' interpretations to original sources. The number of findings categorized as unequivocal, credible or not supported were checked. The overall ConQual score was calculated based on dependability and credibility (Aromataris et al. 2024; Munn et al. 2014).

## 3 Results

A total of 628 eligible papers from databases and 77 eligible papers from websites were identified. A total of 298 papers were excluded during title and abstract screening, and 187 papers were excluded during full‐text screening (Supporting file 2). In summary, 18 papers were included after title and abstract screening and full‐text screening. A total of 2143 eligible papers were identified from citation searching, of which 915 studies were excluded. Four papers were included after citation searching. Two primary studies were included after the search update.

In total, 24 studies were included (Figure 1). Publication dates range from 2011 to 2025. The studies cover clinical and non‐clinical settings as well as qualitative studies and mixed‐method studies (Supporting file 3). The studies address formal, informal, mixed, and unknown types of HME (Table 4, Supporting file 3). All populated continents are represented (Figure 2) (Ahmed et al. 2024; Alves et al. 2013; Bressler et al. 2020; Chugh Sachdeva et al. 2019; Coutsoudis et al. 2011; Esquerra‐Zwiers et al. 2016; Faruqui et al. 2023; Gelano et al. 2018; Gribble 2013; Gribble 2014; Karacan et al. 2024; Karami et al. 2025; Kürtüncü et al. 2024; Loh et al. 2023; Lubbe et al. 2019; Mackenzie et al. 2013; Magowan et al. 2020; Mampane and Wolvaardt 2024; Mondkar et al. 2018; O'Sullivan et al. 2016; Oreg and Negev 2023; Oueidat et al. 2020; Rabinowitz et al. 2018; Vesel et al. 2023). The findings reflect the included studies rather than continent‐wide cultural patterns. It should be highlighted that one study focuses on an Ultra‐Orthodox (Haredi) Jewish Community (Bressler et al. 2020).

**Figure 1:**
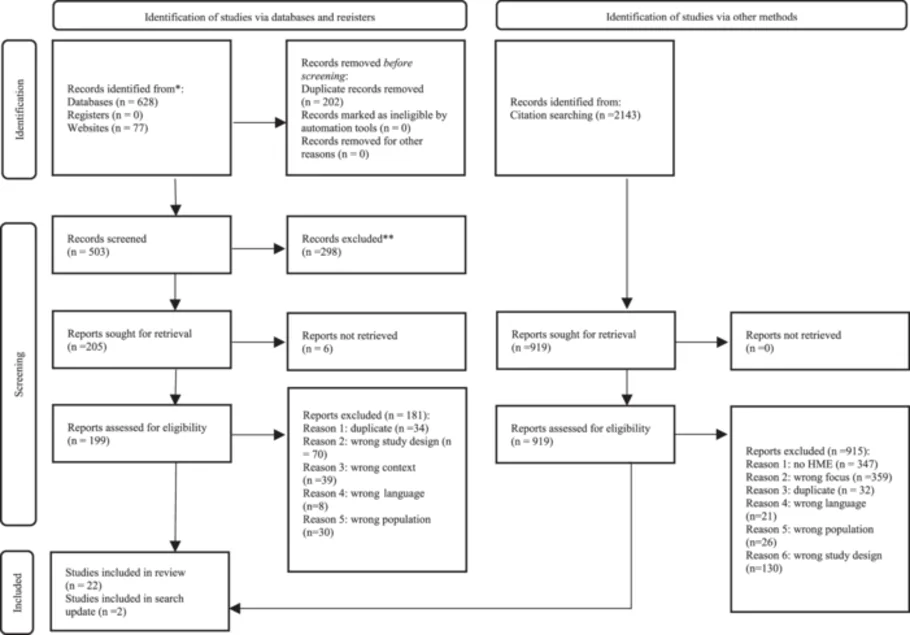
PRISMA diagram of search strategy (Page et al. 2021).

**Figure 2:**
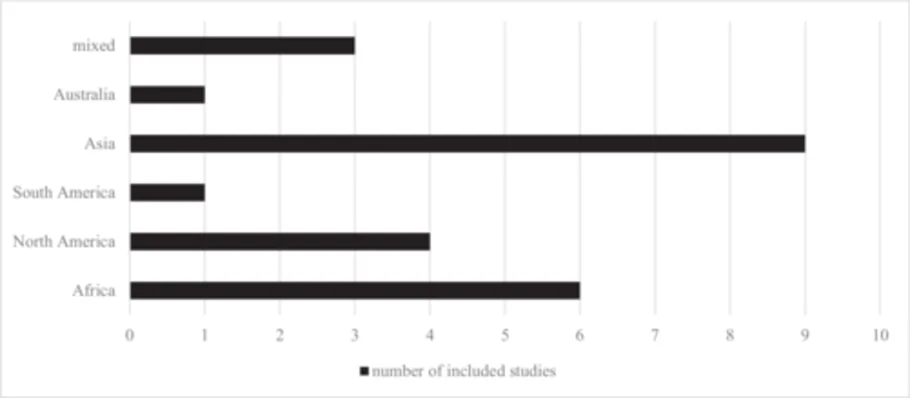
Continents. Mixed refers to studies conducted in North America, Europe, Australia, Asia, and Africa.

The critical appraisal results range from 6 to 9 (Table 2). No paper was excluded on the grounds of methodological quality scoring (Aromataris et al. 2024; Lockwood et al. 2015).

**Table 3:** ConQual summary of findings (Munn et al. 2014).

Systematic review title: Parents' transcultural attitudes and knowledge concerning human milk exchange: A qualitative systematic review and meta‐aggregation Population: (expectant) parents until two years postpartum Phenomena of Interest: religion‐ and culture‐related attitudes and knowledge towards HME Context: clinical and non‐clinical setting				
Synthesized finding	Type of research	Dependability	Credibility	ConQual score
1. Religion	58,33% qualitative studies	unchanged	Downgraded one	Moderate
2. Culture	88,89% qualitative studies	unchanged	Downgraded one	Moderate
3. Dyad composition	75% qualitative studies	unchanged	Downgraded one	Moderate
4. Network	81,25% qualitative studies	unchanged	Downgraded one	Moderate

*Note:* Dependability: unchanged (4–5 ‘yes’ responses answering the five question of JBI‐QARI), downgraded one (2–3 ‘yes’ responses answering the five question of JBI‐QARI), downgraded two (0‐1 ‘yes’ responses answering the five question of JBI‐QARI).

Credibility: unchanged (unequivocal findings), downgraded one (mix of unequivocal and credible findings), downgraded three (credible/not supported findings).

ConQual score: level of confidence depending on dependability and credibility: high (unchanged), moderate (downgraded one), low (downgraded two) and very low (downgraded three).

Data extraction was conducted on general data (Supporting file 3) and data relevant to answering the research question. A total of 162 findings were coded (Supporting file 4), with 21 findings not incorporated into the synthesis on the basis of unsupported credibility indicators. In the data synthesis process, 16 categories were inductively formed, and four synthesized findings were identified (Figure 3) (Aromataris et al. 2024). All synthesized findings achieved a moderate ConQual score (Table 3) (Aromataris et al. 2024; Munn et al. 2014).

**Figure 3:**
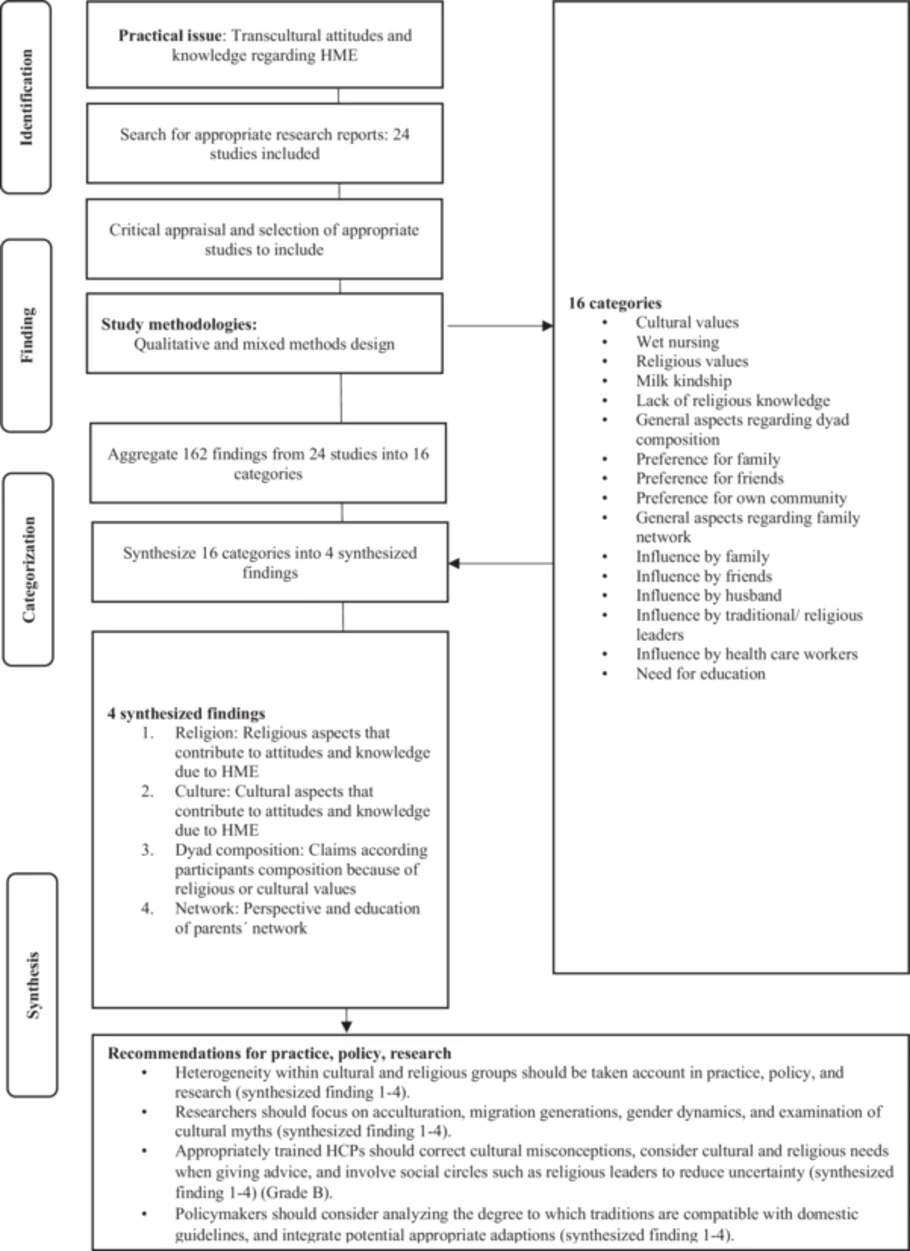
PRISMA diagram of data synthesis (Aromataris et al. 2024; Page et al. 2021).

### 3.1 Religion

The *Religion* synthesized finding includes the categories *Religious values, Milk kinship*, and *Lack of religious knowledge*. Religious values were identified as a contributing factor to positive and negative attitudes towards HME (Alves et al. 2013; Faruqui et al. 2023; Gelano et al. 2018; Gribble 2014; Karacan et al. 2024; Karami et al. 2025; Kürtüncü et al. 2024; Loh et al. 2023; Mondkar et al. 2018; Oreg and Negev 2023; Oueidat et al. 2020). With reference to positive attitudes, parents who participated in a study in South America described the religious significance of milk donation: “To donate is love, it is faith, (…)” (Alves et al. 2013, p. 1172) (credibility: credible). They also highlight the religious value of human milk: “Our milk is sacred” (Alves et al. 2013, p. 1173) (credibility: credible). Milk sharing is considered valuable by religious leaders, with parents in an Asian study saying: “we all are Adam's children… if we help each other, then Almighty Allah will also bless us with his mercy…” (Faruqui et al. 2023, p. 88) (credibility: unequivocal). On the side of negative attitudes, religious traditions are described as barriers, for example, when parents from Lebanon explain: “not allowed in my religion” (Oueidat et al. 2020) (credibility: unequivocal).

For Muslims, milk kinship can be another obstacle (Faruqui et al. 2023). This refers to the belief that a kinship relationship is formed between the milk donor and the recipient child, and it has consequences for future relationships (El‐Khuffash and Unger 2012). As a mother from Iran described it: “Milk makes siblings; I don't want my child tied to a stranger. What if, in the future, my child unknowingly meets and forms a relationship with someone who is their milk sibling?” (Karami et al. 2025, p. 7) (credibility: unequivocal).

On the matter of knowledge, parents indicate uncertainty regarding religious obligations. Parents from Ethiopia asserted: “rather it is better to feed formula or cow milk by bottle” (Gelano et al. 2018, p. 6) (credibility: unequivocal). A mother in an Iran study said: “If (…) religious scholar endorsed it and explained the safety measures, I'd feel more comfortable.” (Karami et al. 2025, p. 9) (credibility: unequivocal).

### 3.2 Culture

The *Culture* synthesized finding contains the categories *Cultural values* and *Wet nursing*. Cultural values were identified as a contributing factor to positive and negative attitudes towards HME (Ahmed et al. 2024; Bressler et al. 2020; Coutsoudis et al. 2011; Karacan et al. 2024; Karami et al. 2025; Lubbe et al. 2019; Mackenzie et al. 2013; Magowan et al. 2020; Mampane and Wolvaardt 2024). On the positive side, informal milk sharing is described as “a respected and valued cultural norm” (Bressler et al. 2020) in a primary study in North America. With regard to negative attitudes, cultural myths exist which negatively influence the decision‐making process on this subject. There are myths about the transmission of intelligence (Lubbe et al. 2019, p. 6) or of personality via human milk, which are especially prevalent in Africa: “There are some genetic issues everything is to do with a DNA thing. Some people fear their babies to feed on the breasts of thieves, murderers, mentally disturbed people and the like that their children will end up like that.” (Ahmed et al. 2024, p. 11) (credibility: unequivocal). Some people even believe that receiving children will die: “Then another thing as Africans we respect totems, in Ankole there is a clan where by their wives can't breastfeed another person's baby because if you do it the child will die.” (Ahmed et al. 2024, p. 11) (credibility: unequivocal).

Wet nursing, or breastfeeding by another mother, is a common cultural practice (Shaikh and Ahmed 2006). Different authors confirm that HME acceptance correlates with awareness about wet nursing: “she left a baby at 2 months and her sister used to breastfeed two babies, and they grew as if they were twins and they are 3 years” (Ahmed et al. 2024, p. 8) (credibility: unequivocal). The bonding is described as a barrier: “I will accept as long as the mother who is donating will not look at my baby in the eyes or face so that the baby will not feel or think that the person is her mother, there must not be a bond between the two.” (Mampane and Wolvaardt 2024, p. 5) (credibility: unequivocal).

In terms of knowledge, wet nursing leads to limited awareness of HMBs. An Australian parent stated: “If I was to explain to my husband what an actual milk bank is… that it is screened and all those sorts of things, I think he'd feel more comfortable about it.” (Mackenzie et al. 2013, p. 225) (credibility: credible). Cultural beliefs which are not grounded in scientific evidence may contribute to misconceptions (Ahmed et al. 2024; Lubbe et al. 2019).

### 3.3 Dyad Composition

The *Dyad composition* synthesized finding consists of the categories G*eneral aspects regarding dyad composition, Preference for family, Preference for friends*, and *Preference for own community*. Irrespective of the type of HME, expectations can arise with respect to the composition of donor and recipient child. Cultural and religious preferences regarding dyad formation demonstrate how attitudes and knowledge shape HME (Bressler et al. 2020; Chugh Sachdeva et al. 2019; Coutsoudis et al. 2011; Faruqui et al. 2023; Gelano et al. 2018; Gribble 2013; Karacan et al. 2024; Karami et al. 2025; Kürtüncü et al. 2024; Lubbe et al. 2019; Mampane and Wolvaardt 2024; O'Sullivan et al. 2016; Oreg and Negev 2023; Rabinowitz et al. 2018; Vesel et al. 2023).

There are different opinions about whether participants should be known. Many parents would want to know each other: “I think that would be too impersonal for me. I like knowing who is getting my breastmilk.” (Gribble 2013, p. 455) (credibility: unequivocal). In some cases, they want to make sure that requirements are fulfilled: “If I knew the donor was a good, religious person, I'd use it. I need to be sure that they follow halal practices (…).” (Karami et al. 2025, p. 8) (credibility: unequivocal). With the consideration of milk kinship, the gender is relevant: “If the donor's child is the same gender as mine, I'd be even more careful” (Karami et al. 2025, p. 8) (credibility: unequivocal); this creates a need for documentation and transparency (Karami et al. 2025). Meanwhile, some authors assert the existence of distrust if the families do not know each other (Coutsoudis et al. 2011).

Another thing is to whom the human milk is shared and from whom the human milk is accepted. On the one hand, a preference for sharing among relatives and friends is evident: “In fact, we can breastfeed our babies by our relatives or family members.” (Faruqui et al. 2023, p. 85) (credibility: unequivocal). Yet in other instances, community members are preferred: “I would do anything for any baby in my community (…).” (Bressler et al. 2020, p. 339) (credibility: unequivocal).

### 3.4 Network

The *Network* synthesized finding comprises the categories *General aspects regarding family network, Influence by family, Influence by friends, Influence by husband, Influence by traditional/religious leaders, Influence by health care workers*, and *Need for education*. The social environments identified constitute the social context in which attitudes and knowledge are developed (Ahmed et al. 2024; Alves et al. 2013; Bressler et al. 2020; Chugh Sachdeva et al. 2019; Coutsoudis et al. 2011; Esquerra‐Zwiers et al. 2016; Faruqui et al. 2023; Gelano et al. 2018; Karacan et al. 2024; Karami et al. 2025; Loh et al. 2023; Lubbe et al. 2019; Mackenzie et al. 2013; Magowan et al. 2020; Mondkar et al. 2018; Vesel et al. 2023). While families talk about strong influence from the social environment (Karacan et al. 2024), many others describe limited influence. Reasons for this are given as a lack of time for discussions (Esquerra‐Zwiers et al. 2016; Mackenzie et al. 2013) or lack of knowledge of the network: “When they learn and see what happens in the NICU, then they will be kind to donate” (Ahmed et al. 2024, p. 12) (credibility: unequivocal).

Different groups have an influence on parents. For one, the opinion of the families is relevant: “Some families tell their daughter‐in‐law not to share their breast milk with others, if they do then they will not tolerate this…” (Faruqui et al. 2023, p. 89) (credibility: unequivocal). Moreover, the husband's permission is important: “If the husband permits, then it's okay to share.” (Faruqui et al. 2023, p. 88) (credibility: unequivocal). Support from friends is also clarified as important (Mackenzie et al. 2013). Leaders are described as both facilitators and barriers: “Some might be young and might not understand these issues well, but traditional leaders I think will encourage us to donate some breast milk.” (Ahmed et al. 2024, p. 12) (credibility: unequivocal). In this context, HCPs were classified as the “main contacts” (Mackenzie et al. 2013, p. 227) (credibility: credible). Across several illustrations, it can be seen that it is ultimately the husband who “decides what's best” (Karami et al. 2025, p. 8) (credibility: unequivocal).

## 4 Discussion

Four synthesized findings were identified and attained a moderate ConQual score. This indicates moderate usefulness and trustworthiness; the results provide relevant insight, but the recommendations should be interpreted with caution (Aromataris et al. 2024; Munn et al. 2014).

There are a variety of religious traditions and cultural values that determine attitudes and knowledge. Culture and religion often determine dyad composition and network priorities (Ahmed et al. 2024; Alves et al. 2013; Bressler et al. 2020; Chugh Sachdeva et al. 2019; Coutsoudis et al. 2011; Esquerra‐Zwiers et al. 2016; Faruqui et al. 2023; Gelano et al. 2018; Gribble 2013; Gribble 2014; Karacan et al. 2024; Karami et al. 2025; Kürtüncü et al. 2024; Loh et al. 2023; Lubbe et al. 2019; Mackenzie et al. 2013; Magowan et al. 2020; Mampane and Wolvaardt 2024; Mondkar et al. 2018; O'Sullivan et al. 2016; Oreg and Negev 2023; Oueidat et al. 2020; Rabinowitz et al. 2018; Vesel et al. 2023).

Instances of cross‐theme integration were noted: The identified networks shape the cultural and religious beliefs which are relevant to acceptance of HME, such as dyad formation. Cultural and religious preferences demonstrate how attitudes and knowledge shape HME. Misconceptions are determinants of trust in HMBs. The allocation of data extracts into religion or culture was based on the same classification used in the primary study; this resulted in a broad categorization of religion, where significant variations in the interpretation of religious beliefs are minimally captured in the studies. The concept of culture itself is also subject to ongoing academic debate, as its usage is often inconsistent (Nünning 2009). Religion often overlaps with the categories that profess a preference for HME to take place among community and family. Religion also intersects with categories concerning the relevance of education, and of husbands' and relatives' opinions. Culture overlaps with the category regarding the importance of community leaders and husband. Culture also intersects with the preference for HME to take place among community. Culture and religion overlap with the need for education within the community (Ahmed et al. 2024; Alves et al. 2013; Bressler et al. 2020; Chugh Sachdeva et al. 2019; Coutsoudis et al. 2011; Esquerra‐Zwiers et al. 2016; Faruqui et al. 2023; Gelano et al. 2018; Gribble 2013; Gribble 2014; Karacan et al. 2024; Karami et al. 2025; Kürtüncü et al. 2024; Loh et al. 2023; Lubbe et al. 2019; Mackenzie et al. 2013; Magowan et al. 2020; Mampane and Wolvaardt 2024; Mondkar et al. 2018; O'Sullivan et al. 2016; Oreg and Negev 2023; Oueidat et al. 2020; Rabinowitz et al. 2018; Vesel et al. 2023).

A comparison between different religious groups is not possible due to insufficient information in the studies. A comparison of studies in community and clinical settings shows that participants in community studies often favor HME among community, while interviews conducted during studies in clinical settings frequently show a preference for family (Ahmed et al. 2024; Alves et al. 2013; Bressler et al. 2020; Chugh Sachdeva et al. 2019; Coutsoudis et al. 2011; Esquerra‐Zwiers et al. 2016; Faruqui et al. 2023; Gelano et al. 2018; Gribble 2013; Gribble 2014; Karacan et al. 2024; Karami et al. 2025; Kürtüncü et al. 2024; Loh et al. 2023; Lubbe et al. 2019; Mackenzie et al. 2013; Magowan et al. 2020; Mampane and Wolvaardt 2024; Mondkar et al. 2018; O'Sullivan et al. 2016; Oreg and Negev 2023; Oueidat et al. 2020; Rabinowitz et al. 2018; Vesel et al. 2023). On the subject of comparing cultural groups, there are underlying attitudes which have accrued even though heterogeneity and dynamics within the cultural groups were considered. The results refer to the included studies rather than continent‐wide cultural values. With regard to the included studies conducted in Africa, many participants justify their attitude with reference to culture (Ahmed et al. 2024; Coutsoudis et al. 2011; Lubbe et al. 2019; Magowan et al. 2020; Mampane and Wolvaardt 2024), with African mythology and the presence of HIV potentially shaping views on HME. Concerning the included studies conducted in Asia, many interviewees ground their attitudes and knowledge in religion (Faruqui et al. 2023; Karacan et al. 2024; Karami et al. 2025; Kürtüncü et al. 2024; Loh et al. 2023; Mondkar et al. 2018; Oreg and Negev 2023; Oueidat et al. 2020); this is perhaps because the Qur'an is the only Abrahamic religious text which contains declarations on breastfeeding (Subudhi and Sriraman 2021).

The diversity within each community should be considered. Insights collected in a specific geographical location do not necessarily reflect the culture of the native people in that place. Some of the participants in studies in North America identify themselves as Asian, Latinx, multiracial (Rabinowitz et al. 2018) or people of color (Esquerra‐Zwiers et al. 2016). Migration status (Schenk et al. 2006) or degree of acculturation (Frankenberg and Bongard 2013) may be useful factors to consider.

The findings are consistent with the current literature. This review also demonstrates that culture and religion are barriers as well as facilitators to acceptance of HME (Mathias et al. 2023). The review also highlights the need for religion‐linked HMBs, particularly Islam‐compliant HMBs on account of beliefs concerning milk kinship (Gribble et al. 2025).

The results are equivalent to theoretical frameworks. The *Theory of planned behavior* posits that attitudes towards a behavior, subjective norm, and perceived behavioral control determine the intention of behavior (Ajzen 1991). Acceptance of HME is affected by attitude, subjective norm (culture, religion, network), and perceived behavioral control (autonomy, knowledge, feasibility of spiritual requirements). These dimensions need to be considered in the course of counselling (Ajzen 1991). The interrelationships serve as proof of the described interaction between the synthesized findings. Misconceptions can result in misinformed decisions: Myths must be investigated and corrected. The *Social Identity Theory* illustrates that affiliation with a community leads to creation of a similar self‐identity that shapes similar beliefs (Tajfel and Turner 1979). It justifies preferences for dyad formation and the relevance of the social environment.

It should be emphasized that no time frame was defined in selecting the studies. The results are relevant to all care settings and diverse healthcare systems because articles from all populated continents were included. ConQual scores were moderate, and as such this review is adjudged as moderately useful (Aromataris et al. 2024; Munn et al. 2014).

The study selection was restricted to papers published in English due to a lack of funding for professional interpreters. A total of 29 studies were excluded on the grounds of foreign language, of which 20 studies also met other exclusion criteria. Nine studies were excluded due to foreign language, of which eight studies relate to South America and one was in Turkey. Findings concerning South America may not be sufficiently represented, and results might disproportionately represent findings from countries in which the English language is the academic norm. Brazil has the most extensive network of HMBs (Pan American Health Organization n.d.), and the same approach has been expanded to many countries in South America (United Nations Development Programme 2020). Findings regarding formal HME may be missed. Moreover, access to full texts was limited, though full texts were requested from all authors of articles with limited access. The search strategy had typographical errors; however, given the comprehensive, sensitive search strategy, the research team does not anticipate this will have any impact on validity. As the search strategy focused on formal HME, terms related to informal HME were not included, though they were recognized as relevant results. Although studies focusing on informal HME were identified despite the focus on formal HME, the possibility that relevant studies were missed cannot be excluded. This fact must be taken into account in both the interpretation and planning studies. This review primarily considers studies focusing on formal HME (Table 4).

**Table 4:** Types of HME.

Study	Type of HME
Ahmed et al. (2024)	Unknown/mixed
Herdy Alves et al. (2013)	Formal
Bressler et al. (2020)	Informal
Chugh Sachdeva et al. (2019)	Unknown/mixed
Coutsoudis et al. (2011)	Unknown/mixed
Esquerra‐Zwiers et al. (2016)	Formal
Faruqui et al. (2023)	Unknown/mixed
Gelano et al. (2018)	Formal
Gribble (2013)	Formal
Gribble (2014)	Informal
Karacan et al. (2024)	Formal
Karami et al. (2025)	Formal
Kürtüncü et al. (2024)	Formal
Loh et al. (2023)	Formal
Lubbe et al. (2019)	Formal
Mackenzie et al. (2013)	Formal
Magowan et al. (2020)	Formal
Mampane and Wolvaardt (2024)	Unknown/mixed
Mondkar et al. (2018)	Unknown/mixed
O´Sullivan et al. (2016)	Informal
Oreg and Negev (2023)	Informal
Oueidat et al. (2020)	Unknown/mixed
Rabinowitz et al. (2018)	Unknown/mixed
Vesel et al. (2023)	Unknown/mixed

There were deviations from the study protocol (CRD42024564218). Use of CAMELOT was planned for quality assessment (Munthe‐Kaas et al. 2024). The JBI Critical Appraisal Checklist for Qualitative Research was used because it is the most coherent (Hannes et al. 2010). More granular subgroup analysis was not possible because of insufficient data. The conceptual framing of transculturality was adapted to highlight the intra‐group diversity (Welsch 1992). The title was adapted to refer to HME so all types were included (Palmquist et al. 2019).

## 5 Conclusions

Parental attitudes and knowledge towards HME are determined by religious beliefs, in particular milk kinship, as well as cultural values, especially myths. Family networks shape attitudes and knowledge. Cultural and religious needs regarding dyad composition express how attitudes and knowledge shape the decision‐making process. All synthesized findings and recommendations were supported by moderate‐confidence evidence and should be interpreted accordingly.

Heterogeneity within communities should be taken into account (synthesized findings 1–4). Researchers should investigate acculturation, migration generations, gender dynamics, and examination of myths, and more prospective studies should be planned. They should summarize their results in evidence‐based information tailored to reflect the diverse backgrounds (synthesized findings 1–4).

Information about cultural and religious beliefs should be incorporated into staff training and education. The decision‐making process needs to bridge the gap between medical necessity and cultural and religious beliefs. HCPs should correct misconceptions, consider traditions, and integrate the perspectives of religious leaders (synthesized finding 1–4) (Grade B).

Policymakers should consider evaluating which traditions are compatible with the regulations in HMBs. There are grounds to reconsider the need for anonymity (synthesized finding 1–4). Milk kinship is anchored in Islam, and anonymity presents an obstacle according to milk kinship (Karacan et al. 2024) which may lead mothers to avoid formal HMBs. Researchers should analyze, whether there is a requirement for needs‐sensitive HMBs, such as Islam‐compliant HMBs.

## Author Contributions

**Julia Will:** conceptualization, methodology, software, validation, formal analysis, resources, data curation, writing – original draft; writing – reviews and editing, visualization, supervision, project administration. **Vivian Kirchhoff:** conceptualization, methodology, validation, formal analysis, writing – reviews and editing. **Julia Lühnen:** conceptualization, methodology, validation, writing – reviews and editing. **Jalid Sehouli:** conceptualization, methodology, validation, writing – reviews and editing. **Antje Tannen:** conceptualization, methodology, validation, formal analysis, writing – reviews and editing.

## Funding

The authors have nothing to report.

## Conflicts of Interest

JW is a member of Frauenmilchbank‐Initiative e.V. JS, VH, JL and AT declare to have no competing interests.

## Supporting information


Supporting File 1

Supporting File 2

Supporting File 3

Supporting File 4

Supporting File 5

## Data Availability

The data that supports the findings of this study are available in the supporting material of this article.
