# Supplementary material for: Parents' Transcultural Attitudes and Knowledge Concerning Human Milk Exchange: A Qualitative Systematic Review and Meta‐Aggregation
Source: Matern Child Nutr. 2026 Oct 27;22(4):e70278. doi: 10.1111/mcn.70278 (PMC13714566; doi:10.1111/mcn.70278)
Supplement: Supplementary file 1 [file MCN-22-e70278-s001.docx]

Supplementary material 2: exclusion criteria full text screening

1. exclusion criteria full text screening (literature search 2024)

| studies | Exclusion criteria |
| --- | --- |
| A, A. (2021). Knowledge and Attitude of Lactating Mothers and Health Professionals Towards Pasteurized Donor Human Milk Banking: A Preliminary Study on the Feasibility of Establishing Donor Human Milk Banking in Neonatal Intensive Care Unit of Selected Hospitals in Addis Ababa. Food Science & Nutrition Technology, 6(2), 1-22. https://doi.org/10.23880/fsnt16000266 | duplicate |
| A, S., K. S, K., V, A., K, P., S, G., & P, S. (2020). A Descriptive Study of the Attitude and Experiences of Donor Mothers in a Human Milk Bank. Journal of Evidence Based Medicine and Healthcare, 7(37), 2005-2010. https://doi.org/10.18410/jebmh/2020/417 | wrong context |
| Abhinav, P., Kultida, S., Lawrence, N., & Jour. (2019). Attitudes towards Donor Breast Milk in an Inner City Population. https://doi.org/doi: | wrong population |
| Adawiah, R., Utomo, M. T., Handayani, K. D., Angelika, D., & Etika, R. (2022). Correlation between Knowledge and Attitude to the Behavior of Health Workers Regarding Acceptance of Human Milk Bank in General Hospital Dr. Soetomo Surabaya. International Journal Of Scientific Advances, 3(1). https://doi.org/10.51542/ijscia.v3i1.20 | wrong population |
| Afonso, V. W., Almeida do Valle, D., de Castro Oliveira Ribeiro, U. R. V., Mussi Monteze, N., Ribeiro, L. C., de Almeida Vargas, A. L., & Monteiro Oliveira, B. (2015). PERFIL DAS USUÁRIAS DE UM BANCO DE LEITE HUMANO, EM JUIZ DE FORA, MG. Revista de Atencao Primaria a Saude, 18(1), 85-91. https://doi.org/doi: | wrong context |
| Agiel, M. T., & Adji Pratama, P. (2022). Breast Milk Donor in Islamic Law Perspective: A Study at The Lactashare Foundation Lowokwaru Malang. Proceeding of Saizu International Conference on Transdisciplinary Religious Studies, 232-240. https://doi.org/10.24090/icontrees.2022.248 | wrong context |
| Akma Jamil, N., Khuan, L., Theng Cheong, A., & Muda, S. M. (2021). An emerging trend in infant feeding practice: a scoping review on breastmilk sharing. Central European Journal of Nursing and Midwifery, 12(4), 555-568. https://doi.org/10.15452/cejnm.2020.11.0036 | wrong study design |
| Al Naqeeb, N., Tolba, A., Elhassanin, A. F., Adel Ata, S., & Azab, A. (2021). Ambulatory Human Milk Donors: An Innovative Solution for Human Milk Banking in Muslim Countries. J Hum Lact, 37(4), 730-735. https://doi.org/10.1177/08903344211036433 | wrong study design |
| Alemu, M., Gashu, D., & Abera, A. (2021). Knowledge and attitude of lactating mothers and health professionals towards pasteurized donor human milk banking: A preliminary study on the feasibility of establishing donor human milk banking in neonatal intensiv care unit of selsected hospitals in Addis Ababa. Food Science & Nutrition Technology, 6(2). https://doi.org/doi: 10.23880/fsnt-16000266 | wrong study design |
| Alhreashy, F. (2020). The Academy of Breastfeeding Medicine 25th Annual International Meeting Online November 5–7, 2020. Breastfeeding Medicine, 15(10), A-1-A-34. https://doi.org/10.1089/bfm.2020.29162.abstracts | wrong context |
| Alnakshabandi, K., & Fiester, A. (2016). Creating religiously compliant milk banks in the Muslim world: a commentary. Paediatr Int Child Health, 36(1), 4-6. https://doi.org/10.1080/20469047.2015.1110336 | wrong study design |
| al-Naqeeb, N. A., Azab, A., Eliwa, M. S., & Mohammed, B. Y. (2000). The introduction of breast milk donation in a Muslim country. J Hum Lact, 16(4), 346-350. https://doi.org/10.1177/089033440001600412 | wrong study design |
| al-Naqeeb, N. A., Azab, A., Eliwa, M. S., & Mohammed, B. Y. (2000). The introduction of breast milk donation in a Muslim country. J Hum Lact, 16(4), 346-350. https://doi.org/10.1177/089033440001600412 | duplicate |
| Anilkumar Khatri, J., Vartak, S., Deshmukh, I., & Avinash Kinikar, A. (2023). A Descriptive Study of the Attitude and Experiences of Donor Mothers in a Human Milk Bank at a Tertiary Healthcare Centre. International Journal of Advanced Research, 11(03), 1316-1323. https://doi.org/10.21474/ijar01/16569 | wrong study design |
| Asai, H., Mizuno, K., Yamashita, K., Samejima, M., Yamamoto, K., Hawthorne, K. M., Furukawa, K., Hinokuma, N., & Otsuka, N. (2020). Survey of neonatal ICU nurses’ knowledge of neonatal nutrition in Japan. Journal of Neonatal Nursing, 26(1), 57-58. https://doi.org/10.1016/j.jnn.2019.08.001 | wrong population |
| Aysun, E., Yeşim, Y., & Esin Turfan, Ç. (2015). Annelerin süt bankası hakkındaki görüşleri: İzmir örneği ; Mothers’ views of milk banking: sample of İzmir. Turkish Archives of Pediatrics, 50(2), 83-89. https://doi.org/DOI: 10.5152/tpa.2015.2406 | wrong study design |
| Azad, M. B., Nickel, N. C., Bode, L., Brockway, M., Brown, A., Chambers, C., Goldhammer, C., Hinde, K., McGuire, M., Munblit, D., Patel, A. L., Perez-Escamilla, R., Rasmussen, K. M., Shenker, N., Young, B. E., & Zuccolo, L. (2021). Breastfeeding and the origins of health: Interdisciplinary perspectives and priorities. Matern Child Nutr, 17(2), e13109. https://doi.org/10.1111/mcn.13109 | wrong context |
| Azema, E., & Callahan, S. (2003). Breast milk donors in France: a portrait of the typical donor and the utility of milk banking in the French breastfeeding context. J Hum Lact, 19(2), 199-202. https://doi.org/10.1177/0890334403252476 | wrong study design |
| Bai, Y., & Kuscin, J. (2021). The Current State of Donor Human Milk Use and Practice. J Midwifery Womens Health, 66(4), 478-485. https://doi.org/10.1111/jmwh.13244 | wrong study design |
| Bataitytė, T. (2020). Nėščiųjų žinios ir požiūris į donorinį motinos pieną ir donorinį motinos pieno banką ; Pregnant Woman Knowledge and Approach to Breastfeeding and Breast Milk Donor Bank. https://doi.org/doi: | no fulltext |
| Baumgartel, K. L., Sneeringer, L., & Cohen, S. M. (2016). From royal wet nurses to Facebook: The evolution of breastmilk sharing. Breastfeed Rev, 24(3), 25-32. https://doi.org/doi: | wrong study design |
| Beevi S S, S. (2021). Assessment of Knowledge regarding Human Breast Milk Bank among the Nursing Officers in JIPMER Puducherry. Medicon Medical Sciences, 1(2). https://doi.org/doi: | wrong population |
| Belfort, M. B., Drouin, K., Riley, J. F., Gregory, K. E., Philipp, B. L., Parker, M. G., & Sen, S. (2018). Prevalence and Trends in Donor Milk Use in the Well-Baby Nursery: A Survey of Northeast United States Birth Hospitals. Breastfeed Med, 13(1), 34-41. https://doi.org/10.1089/bfm.2017.0147 | wrong population |
| Benevenuto de Oliveira, M. M., & Silva, I. A. (2020). Representações sociais de doadoras sobre a doação de leite humano em um hospital universitário. Ciencia, Cuidado e Saude, 19, 1-9. https://doi.org/10.4025/ciencuidsaude.v19i0.47104 | wrong context |
| Bertino, E., Rossi, C., Di Nicola, P., Peila, C., Maggiora, E., Vagliano, L., & Coscia, A. (2015). New perspectives in human milk banks. https://doi.org/doi: | wrong context |
| Bhoola, P., & Biggs, C. (2021). Factors Affecting the Decision of Postnatal Mothers to Donate Milk at a Government Satellite Human Milk Bank Site, in KwaZulu Natal, South Africa. J Hum Lact, 37(1), 95-104. https://doi.org/10.1177/0890334420982915 | wrong study design |
| Brito, R., Mello, T., Santos, D., Lima, A., & Nóbrega, E. (2014). Knowledge of health professionals about the distribution of pasteurized human milk. Revista de Pesquisa: Cuidado é Fundamental Online, 6(1), 261-270. https://doi.org/10.9789/2175-5361.2014v6n1p261 | wrong population |
| Brod, F. R., Rocha, D. L. B., & Santos, R. P. d. (2016). Saberes e práticas de mães de recém-nascidos prematuros perante a manutenção do aleitamento materno Knowledge and practices of mothers of premature newborns in the mainteining of breastfeeding. Revista de Pesquisa Cuidado é Fundamental Online, 8(4), 5108-5113. https://doi.org/10.9789/2175-5361.2016.v8i4.5108-5113 | wrong context |
| Buges, N. M., Klinger, K. d. S. A., & Pereira, R. J. (2020). New mothers and their understanding about breast milk donation. Revista Brasileira de Saúde Materno Infantil:, 20(1). https://doi.org/doi: 10.1590/1806-93042020000100012 | wrong context |
| Çalik Bağriyanik, B., Pekyi̇ği̇t, A., Yildiz, D., & Fidanci, B. (2019). Mothers' Views on Donor Milk and Human Milk Banks. 17, 325-336. https://doi.org/doi: | duplicate |
| Carroll, K., & Herrmann, K. (2012). Introducing donor human milk to the NICU: lessons for Australia. Breastfeed Rev, 20(3), 19-26. https://doi.org/doi: | wrong population |
| Celik, N., Karaca, B., Celik, B., Bereket, N., & Korkmaz, S. (2022). Evaluation of North Syrian Women Knowledge, Opinions and Attitudes Regarding Milk Banks and Milk Donation. Konuralp Tip Dergisi, 14(2), 366-372. https://doi.org/10.18521/ktd.1082661 | wrong study design |
| ÇElİK, N., Karaca, B., ÇElİK, B., Bereket, N., & Korkmaz, S. (2022). Evaluation of North Syrian Women Knowledge, Opinions and Attitudes Regarding Milk Banks and Milk Donation ; Kuzey Suriyeli Kadınların Süt Bankaları ve Süt Bağışına İlişkin Bilgi, Görüş ve Tutumlarının Değerlendirilmesi. Konuralp Medical Journal 14(2), 366-372. https://doi.org/doi: | duplicate |
| Cevese, R. (2020). “Who Knows if One Day, in the Future, They Will Get Married …?”: Breastmilk, Migration, and Milk Banking in Italy. In A. E. T. Tanya Cassidy (Ed.), Ethnographies of Breastfeeding. Routledge. https://doi.org/doi: 10.4324/9781003085294-7 | wrong study design |
| Chagwena, D. T., Mugariri, F., Sithole, B., Mataga, S. F., Danda, R., Matsungo, T. M., & Maponga, C. C. (2020). Acceptability of donor breastmilk banking among health workers: a cross-sectional survey in Zimbabwean urban settings. Int Breastfeed J, 15(1), 37. https://doi.org/10.1186/s13006-020-00283-y | wrong population |
| Chamsi-Pasha, H., & Albar, M. A. (2017). Islamic medical jurisprudence syllabus: A Review in Saudi Arabia. Med J Malaysia, 72(5), 278-281. https://doi.org/doi: | wrong study design |
| Clift-Matthews, V. (2008). Could you breastfeed another woman's child? British Journal of Midwifery, 16(9), 564-564. https://doi.org/10.12968/bjom.2008.16.9.30878 | wrong study design |
| Clouthier, N., Ulrich, C., & Hartman, D. B. (2019). Acceptability of Donor Human Milk in Muslim Populations in Canada. Can J Diet Pract Res, 80(4), 186-189. https://doi.org/10.3148/cjdpr-2019-006 | wrong study design |
| Clouthier, N., Ulrich, C., & Hartman, D. B. (2019). Acceptability of Donor Human Milk in Muslim Populations in Canada. Can J Diet Pract Res, 80(4), 186-189. https://doi.org/10.3148/cjdpr-2019-006 | duplicate |
| Connolly, R. (2022). Sharing the wealth: A mum’s donor experience. Essence: Newsletter of the Australian Breastfeeding Association, 58(1), 13-35. https://doi.org/doi: | no fulltext |
| de Azevedo, M., & Mendes, E. N. W. (2008). Maintenance of lactation: a challenge for hospitalized premature infant's [sic] mothers. Revista Gaucha de Enfermagem, 29(1), 68-75. https://doi.org/doi: | wrong language |
| Demisse, H., Ameya, G., Alemayehu, T., & Shewangashaw, N. (2022). Knowledge and attitudes towards breast milk donation and associated factors among pregnant mothers attending antenatal care at health centers in Addis Ababa, Ethiopia. https://doi.org/doi:10.21203/rs.3.rs-1921575/v1 | wrong study design |
| Doshmangir, L., Naghshi, M., & Khabiri, R. (2019). Factors Influencing Donations to Human Milk Bank: A Systematic Review of Facilitators and Barriers. Breastfeed Med, 14(5), 298-306. https://doi.org/10.1089/bfm.2019.0002 | wrong study design |
| Eksioglu, A., Yesil, Y., & Turfan, E. C. (2015). Mothers' views of milk banking: sample of Izmir. Turk Pediatri Ars, 50(2), 83-89. https://doi.org/10.5152/tpa.2015.2406 | wrong study design |
| Eksioglu, A., Yesil, Y., & Turfan, E. C. (2015). Mothers' views of milk banking: sample of Izmir. Turk Pediatri Ars, 50(2), 83-89. https://doi.org/10.5152/tpa.2015.2406 | duplicate |
| El-Khuffash, A., & Unger, S. (2012). The concept of milk kinship in Islam: issues raised when offering preterm infants of Muslim families donor human milk. J Hum Lact, 28(2), 125-127. https://doi.org/10.1177/0890334411434803 | wrong study design |
| Ellsworth, L., Sturza, J., & Stanley, K. (2021). An Alternative to Mother's Own Milk: Maternal Awareness of Donor Human Milk and Milk Banks. J Hum Lact, 37(1), 62-70. https://doi.org/10.1177/0890334420939549 | wrong study design |
| Ergin, A., & Uzun, S. U. (2018). Turkish Women's Knowledge, Attitudes, and Behaviors on Wet-Nursing, Milk Sharing and Human Milk Banking. Matern Child Health J, 22(4), 454-460. https://doi.org/10.1007/s10995-018-2433-1 | wrong population |
| Espina Jerez, B., Gómez Cantarino, S., Pina Queirós, P. J., & Siles González, J. (2022). La figura de la nodriza y su implicación en el parentesco de leche para la cultura islámica: marco socio-cultural y jurídico. Cultura de los Cuidados, 26(64), 103-121. https://doi.org/10.14198/cuid.2022.64.10 | wrong language |
| Esquerra-Zwiers, A., Rossman, B., Meier, P., Engstrom, J., Janes, J., & Patel, A. (2016). "It's Somebody Else's Milk": Unraveling the Tension in Mothers of Preterm Infants Who Provide Consent for Pasteurized Donor Human Milk. J Hum Lact, 32(1), 95-102. https://doi.org/10.1177/0890334415617939 | duplicate |
| Esquerra-Zwiers, A. L. (2017). Donor Milk: Women's Experiences and Impact on Feeding Outcomes in Premature Infants https://search.ebscohost.com/login.aspx?direct=true&db=cin20&AN=123313471&site=ehost-live | no fulltext |
| Fernandes, P., & Nayak, S. (2020). Knowledge of Antenatal Mothers Regarding Human Milk Banking. Medico-Legal Update, 20(3), 305-312. https://doi.org/doi: | wrong study design |
| Ferrarello, D., Froh, E. B., Hinson, T. D., & Spatz, D. L. (2019). Nurses' Views on Using Pasteurized Donor Human Milk for Hypoglycemic Term Infants. MCN Am J Matern Child Nurs, 44(3), 157-163. https://doi.org/10.1097/NMC.0000000000000525 | wrong population |
| Ferreira Pinheiro Coutinho, A. C., de Oliveira Soares, A. C., & da Silva Fernandes, P. (2014). CONHECIMENTO DAS MÃES SOBRE OS BENEFÍCIOS DO ALEITAMENTO MATERNO À SAÚDE DA MULHER. Journal of Nursing UFPE / Revista de Enfermagem UFPE, 8(5), 1213-1220. https://doi.org/doi:10.5205/reuol.5863-50531-1-ED.0805201415 | duplicate |
| Ferreira Pinheiro Coutinho, A. C., de Oliveira Soares, A. C., & da Silva Fernandes, P. (2014). KNOWLEDGE OF MOTHERS ABOUT THE BENEFITS OF BREASTFEEDING TO WOMEN´S HEALTH. Journal of Nursing UFPE / Revista de Enfermagem UFPE, 8(5), 1213-1220. https://doi.org/doi:10.5205/reuol.5863-50531-1-ED.0805201415 | wrong context |
| Fonseca-Machado, M. D. O., Parreira, B. D. M., Dias, F. A., Costa, N. D. S., Monteiro, J. C. d. S., & Gomes-Sponholz, F. (2013). Caracterização de nutrizes doadoras de um Banco de Leite Human CHARACTERIZATION OF NURSING MOTHERS FROM A HUMAN MILK BANK. Ciencia, Cuidado e Saude, 12(3), 531-540. https://doi.org/10.4025/cienccuidsaude.v12i3.18192 | wrong language |
| Ghaly, M. (2018). Human Milk-Based Industry in the Muslim World: Religioethical Challenges. Breastfeed Med, 13(S1), S28-S29. https://doi.org/10.1089/bfm.2018.29081.mjg | wrong study design |
| Golsanamloo, S., Hosseinzadeh, M., Mirghafourvand, M., Sahebihagh, M. H., M, M. F., & Roshangar, F. (2022). Knowledge and Attitudes Regarding Donated Milk and their Socio-demographic Predictors in Healthcare Providers in Tabriz, 2021: A Cross-sectional Study. Int J Community Based Nurs Midwifery, 10(4), 301-312. https://doi.org/10.30476/IJCBNM.2022.93927.1963 | duplicate |
| Golsanamloo, S., Hosseinzadeh, M., Mirghafourvand, M., Sahebihagh, M. H., M, M. F., & Roshangar, F. (2022). Knowledge and Attitudes Regarding Donated Milk and their Socio-demographic Predictors in Healthcare Providers in Tabriz, 2021: A Cross-sectional Study. Int J Community Based Nurs Midwifery, 10(4), 301-312. https://doi.org/10.30476/IJCBNM.2022.93927.1963 | wrong population |
| Gribble, K. D. (2014). 'A better alternative': why women use peer-to-peer shared milk. Breastfeeding review : professional publication of the Nursing Mothers' Association of Australia, 22(1), 11-21. https://doi.org/doi: | duplicate |
| Gribble, K. D. (2014). "I'm happy to be able to help:" why women donate milk to a peer via Internet-based milk sharing networks. Breastfeed Med, 9(5), 251-256. https://doi.org/10.1089/bfm.2014.0009 | wrong context |
| Grovslien, A., Torng, H. H., Moro, G. E., Simpson, J., & Barnett, D. (2013). International perspectives on donor milk in and beyond the NICU. In J Hum Lact (Vol. 29, pp. 310-312). | wrong study design |
| Grunert, D. (2018). Decision making surrounding human milk donation : attitudes, subjective norms and barriers University of British Columbia]. http://hdl.handle.net/2429/65349 | wrong study design |
| Gürol, A., Özkan, H., & Çelebioğlu, A. (2014). Turkish women's knowledge and views regarding mother's milk banking. Collegian, 21(3), 239-244. https://doi.org/10.1016/j.colegn.2013.05.002 | wrong study design |
| Gutierrez Dos Santos, B., & Perrin, M. T. (2022). What is known about human milk bank donors around the world: a systematic scoping review. Public Health Nutr, 25(2), 312-322. https://doi.org/10.1017/S1368980021003979 | wrong context |
| Haixia, T., Ping, L., Lianlian, Z., Xiaozhen, Q., Shuli, F., & Ziyue, W. (2022). Postpartum women’s views on human milk banking in a city in Southeast China: a cross-sectional survey. International Breastfeeding Journal, 17(3). https://doi.org/doi: 10.1186/s13006-021-00443-8 | wrong study design |
| Halit, H., Bülent, Ü., Erman, A., Coskun Firat, Ö., & Bahar Öztelcan, G. (2023). Knowledge and attitudes of breastfeeding mothers towards breast milk banking. Family Practice & Palliative Care, 8(2), 44-48. https://doi.org/doi: 10.22391/fppc.1179131 | wrong study design |
| Hatice, B., & Hilmiye, A. (2022). Knowledge and Opinions of Premature Infant Mother’s on Human Milk Banks. Journal of Contemporary Medicine, 12(5), 770-776. https://doi.org/doi: 10.16899/jcm.1145494 | wrong study design |
| Hendricks-Munoz, K. D., Darwish, N., Chahin, N., Newman-Lindsay, S., Shaver, L., Dollings, M., Xu, J., & Coleman, V. (2023). Successes of a Focused Mothers' Own Milk (MOM) Program in Counteracting Unintended Effects of a Donor Milk Program on MOM Rates at Discharge. Breastfeed Med, 18(12), 928-933. https://doi.org/10.1089/bfm.2023.0147 | wrong study design |
| Holland, R. (2005). Donor human milk -- part 1: historical, national and international perspectives. MIDIRS Midwifery Digest, 15(4), 537-541. https://doi.org/doi: | wrong context |
| Hosseinzadeh, M., Mirghafourvand, M., Sahebihagh, M., Aghajari, P., Denson, V. L., & Golsanamloo, S. (2023). Donor Breast Milk Banking: Knowledge and Attitudes of Iranian Mothers. J Transcult Nurs, 34(1), 47-58. https://doi.org/10.1177/10436596221125900 | wrong study design |
| Hosseinzadeh, M., Mirghafourvand, M., Sahebihagh, M., Aghajari, P., Denson, V. L., & Golsanamloo, S. (2023). Donor Breast Milk Banking: Knowledge and Attitudes of Iranian Mothers. J Transcult Nurs, 34(1), 47-58. https://doi.org/10.1177/10436596221125900 | duplicate |
| Hsu, H. T., Fong, T. V., Hassan, N. M., Wong, H. L., Rai, J. K., & Khalid, Z. (2012). Human milk donation is an alternative to human milk bank. Breastfeed Med, 7(2), 118-122. https://doi.org/10.1089/bfm.2011.0006 | duplicate |
| Hsu, H. T., Fong, T. V., Hassan, N. M., Wong, H. L., Rai, J. K., & Khalid, Z. (2012). Human milk donation is an alternative to human milk bank. Breastfeed Med, 7(2), 118-122. https://doi.org/10.1089/bfm.2011.0006 | wrong context |
| Huang, C., Han, W., & Fan, Y. (2021). Knowledge and attitude on donation of breast milk in hospitalized mothers. Gac Sanit, 35(3), 213-215. https://doi.org/10.1016/j.gaceta.2019.09.013 | wrong study design |
| Ighogboja, I. S., Olarewaju, R. S., Odumodu, C. U., & Okuonghae, H. O. (1995). Mothers' attitudes towards donated breastmilk in Jos, Nigeria. J Hum Lact, 11(2), 93-96. https://doi.org/10.1177/089033449501100211 | wrong study design |
| Iloh, K. K., Osuorah, C. D., Ndu, I. K., Asinobi, I. N., Obumneme-Anyim, I. N., Ezeudu, C. E., Oluchi, U. M., Anyanwu, O. U., Ekwochi, U., Ogoke, C. C., Ayuk, A. C., & Obu, H. U. (2018). Perception of donor breast milk and determinants of its acceptability among mothers in a developing community: a cross-sectional multi-center study in south-east Nigeria. Int Breastfeed J, 13(1), 47. https://doi.org/10.1186/s13006-018-0189-2 | wrong study design |
| Jahan, Y., Rahman, S., Shamsi, T., & Jour. (2022). Attitudes and Views Concerning Human Milk Banking Among Mothers Residing in a Rural Region of Bangladesh. Journal of Human Lactation, 38(1), 108-117. https://doi.org/doi: 10.1177/08903344211004439 | wrong study design |
| Jahan, Y., Rahman, S., Shamsi, T., & Sm-Rahman, A. (2022). Attitudes and Views Concerning Human Milk Banking Among Mothers Residing in a Rural Region of Bangladesh. Journal of Human Lactation, 38(1), 108-117. https://doi.org/10.1177/08903344211004439 | duplicate |
| Jahan, Y., Rahman, S., Shamsi, T., & Sm-Rahman, A. (2022). Attitudes and Views Concerning Human Milk Banking Among Mothers Residing in a Rural Region of Bangladesh. J Hum Lact, 38(1), 108-117. https://doi.org/10.1177/08903344211004439 | duplicate |
| Kair, L. R., & Flaherman, V. J. (2017). Donor Milk or Formula: A Qualitative Study of Postpartum Mothers of Healthy Newborns. J Hum Lact, 33(4), 710-716. https://doi.org/10.1177/0890334417716417 | wrong context |
| Kair, L. R., Nidey, N. L., Marks, J. E., Hanrahan, K., Femino, L., Fernandez, Y. G. E., Ryckman, K., & Wood, K. E. (2020). Disparities in Donor Human Milk Supplementation Among Well Newborns. J Hum Lact, 36(1), 74-80. https://doi.org/10.1177/0890334419888163 | wrong study design |
| Karadag, A., Ozdemir, R., Ak, M., Ozer, A., Dogan, D. G., & Elkiran, O. (2015). Human milk banking and milk kinship: Perspectives of mothers in a Muslim country. J Trop Pediatr, 61(3), 188-196. https://doi.org/10.1093/tropej/fmv018 | wrong study design |
| Kassierer, M. Y., O'Connor, D. L., Rutherford, E., Rolnitzky, A., & Unger, S. (2014). Implications for observant Jewish families in the provision of mother's own and donor milk for their very low birth weight infant. J Hum Lact, 30(4), 402-404. https://doi.org/10.1177/0890334414545538 | wrong study design |
| Kaur, M., Raghuvanshi, S., & Kang, H. K. (2019). Knowledge and Attitude of Indian Parous Women toward Human Milk Banking. Indian J Community Med, 44(2), 175-176. https://doi.org/10.4103/ijcm.IJCM_377_18 | wrong population |
| Keim, S. A., McNamara, K. A., Dillon, C. E., Strafford, K., Ronau, R., McKenzie, L. B., & Geraghty, S. R. (2014). Breastmilk sharing: awareness and participation among women in the Moms2Moms Study. Breastfeed Med, 9(8), 398-406. https://doi.org/10.1089/bfm.2014.0032 | wrong study design |
| KİLcİ ErcİYas, Ş., ÖZsavran, M., & Ayyildiz, T. (2021). Views of Postnatal Mothers Regarding Human Milk Banking ; Yeni Doğum Yapmış Annelerin Anne Sütü Bankacılığına İlişkin Düşünceleri. Medical Journal of Western Black Sea, 5(3). https://doi.org/doi: 10.29058/mjwbs.893944 | wrong study design |
| Kimani-Murage, E. W., Wanjohi, M. N., Kamande, E. W., Macharia, T. N., Mwaniki, E., Zerfu, T., Ziraba, A., Muiruri, J. W., Samburu, B., Govoga, A., Kiige, L. W., Ngwiri, T., Mirie, W., Musoke, R., Amundson-Mansen, K., & Israel-Ballard, K. (2019). Perceptions on donated human milk and human milk banking in Nairobi, Kenya. Matern Child Nutr, 15(4), e12842. https://doi.org/10.1111/mcn.12842 | duplicate |
| Kimani-Murage, E. W., Wanjohi, M. N., Kamande, E. W., Macharia, T. N., Mwaniki, E., Zerfu, T., Ziraba, A., Muiruri, J. W., Samburu, B., Govoga, A., Kiige, L. W., Ngwiri, T., Mirie, W., Musoke, R., Amundson-Mansen, K., & Israel-Ballard, K. (2019). Perceptions on donated human milk and human milk banking in Nairobi, Kenya. Matern Child Nutr, 15(4), e12842. https://doi.org/10.1111/mcn.12842 | wrong population |
| Kimani-Murage, E. W., Wanjohi, M. N., Kamande, E. W., Macharia, T. N., Mwaniki, E., Zerfu, T., Ziraba, A., Muiruri, J. W., Samburu, B., Govoga, A., Kiige, L. W., Ngwiri, T., Mirie, W., Musoke, R., Amundson-Mansen, K., & Israel-Ballard, K. (2019). Perceptions on donated human milk and human milk banking in Nairobi, Kenya. Matern Child Nutr, 15(4), e12842. https://doi.org/10.1111/mcn.12842 | duplicate |
| Klotz, D., Jansent, S., Glanzmanni, R., Haiden, N., Fuchsl, H., & Gebauet, C. (2021). Donor Human Milk Programs In German, Austrian And Swiss Neonatal Units - Findings From An International Survey. Neonatal Intensive Care, 34(1), 44-49. https://doi.org/doi: | wrong context |
| Koh, N. Y. T., Chien, C. M., Saffari, S. E., & Samuel, R. V. (2023). Knowledge, attitudes, and perceptions of health-care workers about human milk banking in Singapore. Journal of Clinical Neonatology, 12(1), 22-26. https://doi.org/10.4103/jcn.jcn_99_22 | wrong population |
| Konukbay, D., Kapan, E., & Yildiz, D. (2023). Determining Knowledge and Opinions of Neonatal Intensive Care Unit Nurses on Wet-Nursing and Human Milk Banks: A Cross-Sectional Study in Turkiye. J Hum Lact, 39(4), 636-647. https://doi.org/10.1177/08903344231196559 | wrong population |
| Kundisova, L., Bocci, G., Golfera, M., Alaimo, L., & Nante, N. (2019). A systematic review of literature regarding the characteristics and motivations of breastmilk donors. Breastfeeding Review, 27(3), 29-42. https://doi.org/doi: | wrong context |
| Lagos Mendoza, S. L., Lombo Caicedo, J. C., Soto Morales, A. M., & Sánchez Rubio, L. (2022). Donar leche humana salva vidas: percepciones de mujeres donantes y receptoras de leche en un banco de leche humana en Colombia. Cultura de los Cuidados, 26(64), 122-135. https://doi.org/10.14198/cuid.2022.64.11 | wrong language |
| Lam, E. Y., Kecskes, Z., & Abdel-Latif, M. E. (2012). Breast milk banking: current opinion and practice in Australian neonatal intensive care units. J Paediatr Child Health, 48(9), 833-839. https://doi.org/10.1111/j.1440-1754.2012.02530.x | wrong population |
| Lam, E. Y., Kecskes, Z., & Abdel-Latif, M. E. (2012). Breast milk banking: current opinion and practice in Australian neonatal intensive care units. J Paediatr Child Health, 48(9), 833-839. https://doi.org/10.1111/j.1440-1754.2012.02530.x | duplicate |
| Magowan, S., Burgoine, K., Ogara, C., Ditai, J., & Gladstone, M. (2020). Exploring the barriers and facilitators to the acceptability of donor human milk in eastern Uganda - a qualitative study. Int Breastfeed J, 15(1), 28. https://doi.org/10.1186/s13006-020-00272-1 | duplicate |
| Mahlatjie, M. A., Bopape, M., & Mothiba, T. M. (2022). Rural Nurses' Views on Breastmilk Banking in Limpopo Province, South Africa: A Qualitative Study. Nurs Reports, 12(4), 747-757. https://doi.org/10.3390/nursrep12040074 | wrong population |
| Marchiori, G. R. S., Alves, V. H., Rodrigues, D. P., Santos, M. V. d., Branco, M. B. L. R., & Gabriel, A. D. (2018). Saberes Sobre Processo De Enfermagem No Banco De Leite Humano. Texto & Contexto - Enfermagem, 27(2), 1-10. https://doi.org/10.1590/0104-070720180000390016 | wrong language |
| Marshall, J. (2024). Expectant Mothers' Knowledge, Perceptions, Experiences, and Acceptance of Human Donor Milk: A Pilot Study digitalcommons.memphis.edu]. https://digitalcommons.memphis.edu/etd/1100/ | wrong study design |
| McCloskey, R. J., & Karandikar, S. (2018). A Liberation Health Approach to Examining Challenges and Facilitators of Peer-to-Peer Human Milk Sharing. J Hum Lact, 34(3), 438-447. https://doi.org/10.1177/0890334418771301 | wrong context |
| McCloskey, R. J., & Karandikar, S. (2019). Peer-to-Peer Human Milk Sharing: Recipient Mothers' Motivations, Stress, and Postpartum Mental Health. Breastfeed Med, 14(2), 88-97. https://doi.org/10.1089/bfm.2018.0182 | wrong context |
| McNally, D., & Spatz, D. L. (2020). Mothers Who Engage in Long-Term Informal Milk Sharing. MCN Am J Matern Child Nurs, 45(6), 338-343. https://doi.org/10.1097/NMC.0000000000000660 | wrong study design |
| Mduma, D. E., & Harbauer, T. (2023, 2023). Knowledge and acceptability towards human donor milk among women attending at Haydom Lutheran Hospital, Tanzania. First Steps of establishing a Human Milk Banc in a rural hospital in Tanzania Zeitschrift fur Geburtshilfe und Neonatologie, http://ovidsp.ovid.com/ovidweb.cgi?T=JS&PAGE=reference&D=emexa&NEWS=N&AN=642215360 | no fulltext |
| Meneses, T. M. X., Oliveira, M. I. C., & Boccolini, C. S. (2017). Prevalence and factors associated with breast milk donation in banks that receive human milk in primary health care units. J Pediatr (Rio J), 93(4), 382-388. https://doi.org/10.1016/j.jped.2016.09.004 | wrong study design |
| Merlino-Barr, S., & Groh-Wargo, S. (2019). Donor Breast Milk for the Preterm Infant: Your Questions Answered! Neonatal Netw, 38(1), 7-16. https://doi.org/10.1891/0730-0832.38.1.7 | wrong study design |
| Michael, G., Antunes, M., Shaik, S., & ... (2024). Health practitioners knowledge, beliefs, and attitudes regarding the use of donor human milk in neonatal intensive care. Maternal and Pediatric Nutrition Journal, 2(2). https://doi.org/doi: 10.4172/mpn.1000108 | wrong population |
| Mondkar, J., Chugh Sachdeva, R., Shanbhag, S., Khan, A., Manuhar Sinha, M., Dasgupta, R., Israel-Ballard, K., & Sabharwal, V. (2018). Understanding Barriers and Facilitators for Human Milk Banking Among Service Providers, Mothers, and Influencers of Preterm and Sick Neonates Admitted at Two Health Facilities in a Metropolitan City in India. Breastfeed Med, 13(10), 694-701. https://doi.org/10.1089/bfm.2018.0103 | duplicate |
| Murray, L., Anggrahini, S. M., Woda, R. R., Ayton, J. E., & Beggs, S. (2016). Exclusive Breastfeeding and the Acceptability of Donor Breast Milk for Sick, Hospitalized Infants in Kupang, Nusa Tenggara Timur, Indonesia: A Mixed-Methods Study. J Hum Lact, 32(3), 438-445. https://doi.org/10.1177/0890334416649152 | wrong context |
| Murray, L., Anggrahini, S. M., Woda, R. R., Ayton, J. E., & Beggs, S. (2016). Exclusive Breastfeeding and the Acceptability of Donor Breast Milk for Sick, Hospitalized Infants in Kupang, Nusa Tenggara Timur, Indonesia: A Mixed-Methods Study. J Hum Lact, 32(3), 438-445. https://doi.org/10.1177/0890334416649152 | duplicate |
| Namuddu, M. G., Kiguli, J., Nakibuuka, V., Nantale, R., & Mukunya, D. (2023). Acceptability of donated breast milk among pregnant women in selected hospitals in central Uganda: a cross-sectional study. Int Breastfeed J, 18(1), 32. https://doi.org/10.1186/s13006-023-00569-x | wrong study design |
| Nangia, S., Sachdeva, R. C., & Sabharwal, V. (2018). Human Milk Banking: An Indian Experience. NeoReviews, 19(4), e201-e210. https://doi.org/10.1542/neo.19-4-e201 | wrong study design |
| Normadiah, D., Mohamad Sabri, H., Nadhirah, N., Zurita Mohd, Y., Zuliza, K., Wan Pauzi Wan, I., & Jour. (2017). The Views of Fuqaha on the Permissibility and Prohibition of Milk Bank. International Journal of Academic Research in Business and Social Sciences, 7(4). https://doi.org/doi: 10.6007/IJARBSS/v7-i4/2801 | wrong population |
| Normadiah Daud, Nadhirah Nordin, Z. M. Y., Wan Pauzi Wan Ibrahim, Zuliza Mohd Kusrin, Sabri , & Haron, N. R., Nurhidayah Ashar. (2017). Syariah-Compliant Milk Bank According to the Opinions of Early and Contemporary Islamic Scholars. International Journal of Academic Research in Business and Social Sciences, 7(5). https://doi.org/doi: 10.6007/IJARBSS/v7-i5/2987 | wrong population |
| Oda, A., & Mizuno, K. (2022). Questionnaire survey on donor human milk programs targeting NICUs in Japan. Pediatr Int, 64(1), e15344. https://doi.org/10.1111/ped.15344 | wrong context |
| Ogundele, T., Ogundele, O., & Bello, E. (2024). Knowledge and perception of mothers toward donor milk and human milk banking: Experience from two centers in Southwest, Nigeria. Nigerian Journal of General Practice, 20(1). https://doi.org/doi: 10.4103/njgp.njgp_18_21 | wrong study design |
| Olsson, E., Diderholm, B., & Blomqvist, Y. T. (2021). "Paying it Forward" - Swedish Women's Experiences of Donating Human Milk. J Hum Lact, 37(1), 87-94. https://doi.org/10.1177/0890334420979245 | wrong context |
| Onat, G., & Karakoc, H. (2019). Informal Breast Milk Sharing in a Muslim Country: The Frequency, Practice, Risk Perception, and Risk Reduction Strategies Used by Mothers. Breastfeed Med, 14(8), 597-602. https://doi.org/10.1089/bfm.2019.0027 | wrong study design |
| Osbaldiston, R., & Mingle, L. A. (2007). Characterization of human milk donors. J Hum Lact, 23(4), 350-357; quiz 358-361. https://doi.org/10.1177/0890334407307547 | wrong context |
| O'Sullivan, E. J., Geraghty, S. R., & Rasmussen, K. M. (2016). Informal Human Milk Sharing: A Qualitative Exploration of the Attitudes and Experiences of Mothers. J Hum Lact, 32(3), 416-424. https://doi.org/10.1177/0890334416651067 | duplicate |
| O'Sullivan, E. J., Geraghty, S. R., & Rasmussen, K. M. (2018). Awareness and prevalence of human milk sharing and selling in the United States. Matern Child Nutr, 14 Suppl 6(Suppl 6), e12567. https://doi.org/10.1111/mcn.12567 | wrong context |
| Özdemir, S., Aksoy, S. D., & Akdag, G. S. (2022). Turkish Women's Beliefs Concerning Human Milk Banking. Journal of Human Lactation, 38(4), 771-779. https://doi.org/Artn 08903344211037989 | wrong population |
| Ozdemir, S., Dilek Aksoy, S., & Soyaslan Akdag, G. (2022). Turkish Women's Beliefs Concerning Human Milk Banking. J Hum Lact, 38(4), 771-779. https://doi.org/10.1177/08903344211037989 | duplicate |
| Ozdemir, S., Dilek Aksoy, S., & Soyaslan Akdag, G. (2022). Turkish Women's Beliefs Concerning Human Milk Banking. J Hum Lact, 38(4), 771-779. https://doi.org/10.1177/08903344211037989 | wrong population |
| ÖZtelcan GÜNdÜZ, B., ÖZkeÇEcİ, C. F., Atas, E., ÜNay, B., & HalİL, H. (2022). Knowledge and attitudes of breastfeeding mothers towards breast milk banking ; Emziren annelerin anne sütü bankacılığına karşı bilgi ve tutumları. https://doi.org/doi: | wrong study design |
| ÖZtelcan GÜNdÜZ, B., ÖZkeÇEcİ, C. F., Atas, E., ÜNay, B., & HalİL, H. (2023). Knowledge and attitudes of breastfeeding mothers towards breast milk banking. FAMILY PRACTICE AND PALLIATIVE CARE, 8(2), 44-48. https://doi.org/10.22391/fppc.1179131 | duplicate |
| Pal, A., Soontarapornchai, K., Noble, L., & Hand, I. (2019). Attitudes towards Donor Breast Milk in an Inner City Population. Int J Pediatr, 2019, 3847283. https://doi.org/10.1155/2019/3847283 | wrong study design |
| Palma, J. A., & Palma, F. (2020). Maternal Breastfeeding or Wet Nursing? Religion, Persecution, and Ideology in the 17th Century. Breastfeed Med, 15(12), 756-758. https://doi.org/10.1089/bfm.2020.0255 | wrong study design |
| Palmquist, A. E., & Doehler, K. (2016). Human milk sharing practices in the U.S. Matern Child Nutr, 12(2), 278-290. https://doi.org/10.1111/mcn.12221 | duplicate |
| Palmquist, A. E., & Doehler, K. (2016). Human milk sharing practices in the U.S. Matern Child Nutr, 12(2), 278-290. https://doi.org/10.1111/mcn.12221 | wrong context |
| Parker, M. G., Barrero-Castillero, A., Corwin, B. K., Kavanagh, P. L., Belfort, M. B., & Wang, C. J. (2013). Pasteurized human donor milk use among US level 3 neonatal intensive care units. J Hum Lact, 29(3), 381-389. https://doi.org/10.1177/0890334413492909 | wrong population |
| Parker, M. G., & Patel, A. L. (2017). Using quality improvement to increase human milk use for preterm infants. Semin Perinatol, 41(3), 175-186. https://doi.org/10.1053/j.semperi.2017.03.007 | wrong study design |
| Patel, N., Sumaraj, P., Gabler, T., Grieve, A., Naidoo, P., Cronin, K., & Jour. (2023). Knowledge of breastfeeding and human milk banks amongst mothers at Chris Hani Baragwanath Academic Hospital: A descriptive study. Wits Journal of Clinical Medicine, 5(1). https://doi.org/doi: 10.18772/26180197.2023.v5n1a4 | wrong study design |
| Pekyiğit, A., Yıldız, D., Eren Fidancı, B., Çalık, B., Dehmen, Ö., Koçak, T., & Altıntaş, S. (2019). Mothers’ Views on Donor Milk and Human Milk Banks. Guncel Pediatri, 17(3), 325-336. https://doi.org/10.4274/jcp.2019.0029 | duplicate |
| Pekyigit, A., Yildiz, D., Fidanci, B. E., Çalik, B., Dehmen, Ö., Koçak, T., & Altintas, S. (2019). Mothers' Views on Donor Milk and Human Milk Banks. Guncel Pediatri-Journal of Current Pediatrics, 17(3), 325-336. https://doi.org/doi:https://dx.doi.org/10.32941/pediatri.647108 | duplicate |
| Pekyiğit, A., Yıldız, D., Fidancı, B. E., Çalık, B., Dehmen, Ö., Koçak, T., & Altıntaş, S. (2019). Annelerin dönor süt ve süt bankalarına ilişkin görüşleri ; Mothers’ views on donor milk and human milk banks. 17(2). https://doi.org/doi: | wrong language |
| Peregoy, J. A., Pinheiro, G. M., Geraghty, S. R., Dickin, K. L., & Rasmussen, K. M. (2022). Human milk-sharing practices and infant-feeding behaviours: A comparison of donors and recipients. Matern Child Nutr, 18(4), e13389. https://doi.org/10.1111/mcn.13389 | wrong context |
| Pereira, C. G., Cardoso, M. V. L., da Silva, G. R. F., & Bezerra, M. G. A. (2008). How and why to be human milk donor? A descriptive study. Online Brazilian Journal of Nursing, 7(3), 1-1. https://doi.org/doi: | wrong language |
| Perez, C. M. (2020). "The Milk is Love": Understanding Mothers' Emotional Attachment to Breastmilk STARS]. https://stars.library.ucf.edu/honorstheses/845 | wrong context |
| Perrin, M. T., Goodell, L. S., Allen, J. C., & Fogleman, A. (2014). A mixed-methods observational study of human milk sharing communities on Facebook. Breastfeed Med, 9(3), 128-134. https://doi.org/10.1089/bfm.2013.0114 | wrong context |
| Perrin, M. T., Goodell, L. S., Fogleman, A., Pettus, H., Bodenheimer, A. L., & Palmquist, A. E. (2016). Expanding the Supply of Pasteurized Donor Milk: Understanding Why Peer-to-Peer Milk Sharers in the United States Do Not Donate to Milk Banks. J Hum Lact, 32(2), 229-237. https://doi.org/10.1177/0890334415627024 | wrong context |
| Perrin, M. T., Goodell, L. S., Fogleman, A., Pettus, H., Bodenheimer, A. L., & Palmquist, A. E. (2016). Expanding the Supply of Pasteurized Donor Milk: Understanding Why Peer-to-Peer Milk Sharers in the United States Do Not Donate to Milk Banks. J Hum Lact, 32(2), 229-237. https://doi.org/10.1177/0890334415627024 | duplicate |
| Pimenteira Thomaz, A. C., Maia Loureiro, L. V., da Silva Oliveira, T., Furtado Montenegro, N. C., Dantas Almeida Junior, E., Fernando Rodrigues Soriano, C., & Calado Cavalcante, J. (2008). The human milk donation experience: motives, influencing factors, and regular donation. J Hum Lact, 24(1), 69-76. https://doi.org/10.1177/0890334407310580 | wrong context |
| Prates, L. A., Schmalfuss, J. M., & Lipinski, J. M. (2015). PROBLEMS AND CONDUCT ADOPTED BY PUERPERAE DURING LACTATION. Journal of Nursing UFPE / Revista de Enfermagem UFPE, 9(2), 500-508. https://doi.org/doi:10.5205/reuol.7028-60723-1-SM.0902201503 | wrong context |
| Rabiatul, A., Martono Tri, U., Kartika Darma, H., Dina, A., & Risa, E. Correlation between Knowledge and Attitude to the Behavior of Health Workers Regarding Acceptance of Human Milk Bank in General Hospital Dr. Soetomo Surabaya. International Journal of Research Publications 89(1), 237-242. https://doi.org/doi: 10.47119/IJRP1008911120212437 | wrong population |
| Rodrigues, L., Abraham, A., Almeida, A., Shaju, A., Jose, A., & Babu, A. (2023). Knowledge and attitude of postnatal mothers towards Human Breast Milk Banking in a selected Hospital. International Journal of Midwifery Nursing and Practices, 1(2). https://doi.org/doi:10.37591/IJMNP | wrong study design |
| Rojjanasrirat, W., Ahmed, A. H., Johnson, R., & Long, S. (2023). Facilitators and Barriers of Human Milk Donation. MCN Am J Matern Child Nurs, 48(5), 273-279. https://doi.org/10.1097/NMC.0000000000000940 | wrong study design |
| Sahin, B. M. (2020). Views of Muslim mothers in Turkey on breast milk donation and human milk banks. Nursing Practice Today, 7(2), 114-120. https://doi.org/doi: | wrong study design |
| Senol, D. K., & Aslan, E. (2017). Women's opinions about human milk donation and human milk banking. Biomedical Research-India, 28(15), 6866-6871. https://doi.org/doi: | wrong study design |
| Senthilkumar, P. (2018). Breast milk donation : acceptance and knowledge of donors and recipients Medical College Hospital ]. http://repository-tnmgrmu.ac.in/9251/ | wrong study design |
| Sharma, K., Kaur, R., Kumar, M., Thakur, P., Sharma, R., Thakur, A., & Devi, A. (2021). Knowledge, Attitude and Perception of Young Women about Human Breast Milk Donation: A study from North India. International Journal of Medical Science and Innovative Research (IJMSIR), 6(3), 59-66. https://doi.org/doi: | wrong population |
| Sharumathy, V., Vetriselvi, P., & Adhisivam, B. (2019). ASSESS THE KNOWLEDGE AND PERCEPTION ON HUMAN BREAST MILK BANK AMONG POSTNATAL MOTHERS. International Journal of Advanced Research, 7(12), 30-35. https://doi.org/doi: 10.21474/IJAR01/10120 | wrong study design |
| Shoghi, M., Nazarshodeh, S., & Borimnejad, L. (2020). Knowledge and Attitude of Nurses Working in a Neonatal Intensive Care Unit on the Use of Human Donor Milk. Journal of Client-centered Nursing Care, 6(1), 55-64. https://doi.org/10.32598/jccnc.6.1.260.2 | duplicate |
| Shoghi, M., Nazarshodeh, S., & Borimnejad, L. (2020). Knowledge and attitude of nurses working in a neonatal intensive care unit on the use of human donor milk. Journal of Client Centered Nursing Care, 6(1), 55-64. https://doi.org/doi: | wrong population |
| Smyk, J. M., Pergol, J., Danielecka, Z., Kacperczyk-Bartnik, J. P., Bartnik, P., Dobrowolska-Redo, A., & Romejko-Wolniewicz, E. (2021). Knowledge, opinions and attitudes of Polish women towards banking and donation of human breast milk. Ginekol Pol, 92(11), 784-791. https://doi.org/10.5603/GP.a2021.0055 | wrong population |
| Sood, N., Kuznetsova, A., & Milanaik, R. (2024, 2024-1-6). Are physicians helping with the donor human milk shortage?: Physicians' self-reported knowledge of breast milk donation and practices recommending https://publications.aap.org/pediatrics/article-abstract/146/1_MeetingAbstract/145/4205 | no fulltext |
| Spano Nakano, A. M., Guerreiro dos Reis, M. C., Bistafa Pereira, M. J., & Gomes, F. A. (2007). Women's social space and the reference for breastfeeding practice. Rev Lat Am Enfermagem, 15(2), 230-238. https://doi.org/10.1590/s0104-11692007000200007 | wrong context |
| Srenivas, A., Kumaravel, K., Anurekha, V., & Prathibha, K. (2020). A Descriptive Study of the Attitude and Experiences of Donor Mothers in a Human Milk Bank. Journal of Evidence Based Medicine and Healthcare, 7(37). https://doi.org/doi: | wrong context |
| Subudhi, S., & Sriraman, N. (2021). Islamic Beliefs About Milk Kinship and Donor Human Milk in the United States. Pediatrics, 147(2). https://doi.org/10.1542/peds.2020-0441 | wrong study design |
| Sunaina, S., & Ragini, S. (2022). Assess the Knowledge Regarding Human Breast Milk Banking Among Women in Selected Rural Area of District Mohali Punjab, With A View to Develop Information Booklet. International Journal of Nursing Education, 14(4), 96-99. https://doi.org/10.37506/ijone.v14i4.18691 | wrong population |
| Suresh Bhakare, S. (2019). Assessing the Effect of Planned Teaching on Knowledge Regarding ' Human Milk Banking' among Staff Nurses in Selected Hospital of Pune City. Nursing Journal of India, CX(05), 210-212. https://doi.org/10.48029/nji.2019.Cx505 | wrong population |
| Szucs, K. A., Axline, S. E., & Rosenman, M. B. (2009). Quintuplets and a mother's determination to provide human milk: it takes a village to raise a baby--how about five? J Hum Lact, 25(1), 79-84. https://doi.org/10.1177/0890334408328385 | wrong study design |
| Thorley, V. (2008). Sharing breastmilk: wet nursing, cross feeding, and milk donations. Breastfeed Rev, 16(1), 25-29. https://doi.org/doi: | wrong study design |
| Thorley, V. (2014). Milk siblingship, religious and secular: History, applications, and implications for practice. Women Birth, 27(4), e16-19. https://doi.org/10.1016/j.wombi.2014.09.003 | wrong context |
| Tian, C., Li, Y., Soowon, L., Xu, Y., Zhu, Q., & Zhao, H. (2021). Lactating Women's Knowledge and Attitudes About Donor Human Milk in China. J Hum Lact, 37(1), 52-61. https://doi.org/10.1177/0890334420939057 | duplicate |
| Tian, C., Li, Y., Soowon, L., Xu, Y., Zhu, Q., & Zhao, H. (2021). Lactating Women's Knowledge and Attitudes About Donor Human Milk in China. J Hum Lact, 37(1), 52-61. https://doi.org/10.1177/0890334420939057 | wrong study design |
| Tian, C., Li, Y., Soowon, L., Xu, Y., Zhu, Q., & Zhao, H. (2021). Lactating Women's Knowledge and Attitudes About Donor Human Milk in China. J Hum Lact, 37(1), 52-61. https://doi.org/10.1177/0890334420939057 | duplicate |
| Tilayie Feto, G., Yadeta Dessie, B., Nega, A., Aboma, M., Aklilu Abrham, R., Yohanes, A., & Fikirte, T. (2018). Acceptability of donor breast milk banking, its use for feeding infants, and associated factors among mothers in eastern Ethiopia. International Breastfeeding Journal 13(23). https://doi.org/doi: 10.1186/s13006-018-0163-z | duplicate |
| Tran, H. T., Nguyen, T. T., Barnett, D., Weaver, G., Nguyen, O. T. X., Van Ngo, Q., Le, H. T. T., Huynh, L. T., Do, C. T., & Mathisen, R. (2021). Trends and Dynamics in the First Four Years of Operation of the First Human Milk Bank in Vietnam. Nutrients, 13(4), 1107. https://doi.org/10.3390/nu13041107 | wrong context |
| Tran, H. T., Nguyen, T. T., Giang, H. T. N., Huynh, L. T., Barnett, D., Mathisen, R., & Murray, J. C. S. (2021). Factors Associated with the Use of Pasteurized Donor Milk for Healthy Newborns: Experience from the First Human Milk Bank in Vietnam. Nutrients, 13(4), 1151. https://doi.org/10.3390/nu13041151 | wrong study design |
| Tu, H., Li, P., Zhu, L., Quan, X., Fan, S., & Wang, Z. (2022). Postpartum women's views on human milk banking in a city in Southeast China: a cross-sectional survey. Int Breastfeed J, 17(1), 3. https://doi.org/10.1186/s13006-021-00443-8 | wrong study design |
| Varer Akpinar, C., Mandiracioglu, A., Ozvurmaz, S., Adana, F., Koc, N., & Kurt, F. (2022). Attitudes towards human milk banking among native turkish and refugee women residing in a rural region of Turkey: a mixed-methods approach. Int Breastfeed J, 17(1), 74. https://doi.org/10.1186/s13006-022-00516-2 | duplicate |
| Varer Akpinar, C., Mandiracioglu, A., Ozvurmaz, S., Adana, F., Koc, N., & Kurt, F. (2022). Attitudes towards human milk banking among native turkish and refugee women residing in a rural region of Turkey: a mixed-methods approach. Int Breastfeed J, 17(1), 74. https://doi.org/10.1186/s13006-022-00516-2 | wrong population |
| Wagg, A. J., Hassett, A., & Callanan, M. M. (2022). "It's more than milk, it's mental health": a case of online human milk sharing. Int Breastfeed J, 17(1), 5. https://doi.org/10.1186/s13006-021-00445-6 | wrong context |
| Wambach, K., Bateson, T., Matheny, P., & Easter-Brown, K. (2019). A Descriptive Study of the Attitudes, Perceptions, and Experiences of Human Milk Donation. Adv Neonatal Care, 19(6), 441-451. https://doi.org/10.1097/ANC.0000000000000659 | duplicate |
| Wambach, K., Bateson, T., Matheny, P., & Easter-Brown, K. (2019). A Descriptive Study of the Attitudes, Perceptions, and Experiences of Human Milk Donation. Adv Neonatal Care, 19(6), 441-451. https://doi.org/10.1097/ANC.0000000000000659 | wrong context |
| Weaver, G. (1999). Good idea! The milky way: the remarkable history of the Queen Charlotte's and Chelsea Hospital milk bank. MIDIRS Midwifery Digest, 9(4), 497-499. https://doi.org/doi: | wrong study design |
| Weckesser, J. S., Pavsic, J., Skariah, M., & Kahn, K. M. (2019). Expedient Donor Milk Preparation to Increase Use on the Mother-Baby Unit. Journal of Obstetric, Gynecologic & Neonatal Nursing, 48(3), S46-S47. https://doi.org/10.1016/j.jogn.2019.04.080 | wrong study design |
| Wiwanitkit, V. (2016). Introducing Donor Milk in a Neonatal Intensive Care Unit: A Developing Country's Perspective: Correspondence (Vol. 83). Springer Nature. https://doi.org/doi:10.1007/s12098-016-2190-3 | wrong study design |
| Yağmur, S., & Duygu Vefikuluçay, Y. (2023). Knowledge and Opinions of Turkish Parents About the Human Milk Bank. Mediterr Nurs Midwifery, 3(1), 10-17. https://doi.org/doi: 10.4274/MNM.2022.22116 | wrong study design |
| Yotthong, B., & Wattanasit, P. (2022). Relationship between Attitude, Subjective Norm, Perceived Behavioral Control, and Mothers, Intention to Use Donor Human Milk. Princess of Naradhiwas University …. https://doi.org/doi: | wrong language |
| Zahorodnia, O. S., Bila, V. V., Chernyak, I., & Lazo, I. I. (2021). Human milk bank in Ukraine – relities and perspectives. https://doi.org/doi: | wrong context |
| Zamahlubi, H. T., Naidoo, K. L., Khan, F., & Masekela, R. (2023). Knowledge, attitudes and awareness regarding donor breast milk: a cross-sectional study of mothers in a high HIV-prevalent area. South African Journal of Clinical Nutrition. https://doi.org/10.1080/16070658.2023.2189347 | duplicate |
| Zamahlubi, H. T., Naidoo, K. L., Khan, F., & Masekela, R. (2023). Knowledge, attitudes and awareness regarding donor breast milk: a cross-sectional study of mothers in a high HIV-prevalent area. South African Journal of Clinical Nutrition, 1-8. https://doi.org/10.1080/16070658.2023.2189347 | wrong contect |
| Zellner, H. (2019). Question: What is the acceptability and use of donor breast milk among varying cultures and religious practices? Support Line, 41(6), 23-24. https://doi.org/doi: | no fulltext |
| Zhang, N., Li, J. Y., Liu, X. W., Jiang, Y. L., Redding, S. R., & Ouyang, Y. Q. (2020). Factors associated with postpartum women's knowledge, attitude and practice regarding human milk banks and milk donation: A cross-sectional survey. Midwifery, 91, 102837. https://doi.org/10.1016/j.midw.2020.102837 | wrong study design |

2. exclusion criteria full text screening (literature search update 2026)

| studies | Exclusion criteria |
| --- | --- |
| Brown A, Jones S, Griffiths C, Jones W, Weaver G, Shenker N. Understanding modifiable barriers to human milk donation in the United Kingdom. J Hum Nutr Diet. 2025;38(1):e13405. | wrong focus |
| Noble A, Khader Kasasfeh M, Eventov Friedman S, Noble LM. Knowledge and Attitudes of Donor Human Milk of Mothers in the Palestinian Territories and Israel. Breastfeed Med. 2025;20(6):395–401. | wrong population |
