# Supplementary material for: Parents' Transcultural Attitudes and Knowledge Concerning Human Milk Exchange: A Qualitative Systematic Review and Meta‐Aggregation
Source: Matern Child Nutr. 2026 Oct 27;22(4):e70278. doi: 10.1111/mcn.70278 (PMC13714566; doi:10.1111/mcn.70278)
Supplement: Supplementary file 2 [file MCN-22-e70278-s002.docx]

Supplementary material 4: Data extraction II

1. Culture: Cultural aspects that influence attitude due to donor human milk
   a. Cultural values: Cultural values that influence attitude due to donor human milk

| Ahmed et al. 2024 | Finding: ill-intentioned peple may harm the donor; Illustration: “Like some will be asking now they are asking for my breast milk and they take it where or do what with it?” (R5/FGD4, a 30-year-old mother of 3 children). (Ahmed et al. 2024, p. 11); Credibility: Unequivocal |
| --- | --- |
| Ahmed et al. 2024 | Finding: Transmission of criminality such as thef, murder or mental illness; Illustration: “There are some genetic issues everything is to do with a DNA thing. Some people fear their babies to feed on the breasts of thieves, murderers, mentally disturbed people and the like that their children will end up like that." (Ahmed et al. 2024, p. 11); Credibility: Unequivocal |
| Ahmed et al. 2024 | Finding: Fear transmission; Illustration: "Then another thing as Africans we respect totems, in Ankole there is a clan where by their wives can’t breastfeed another person’s baby because if you do it the child will die. In Buganda I hear you don’t eat or move close to the clans or totems.” (Ahmed et al. 2024, p. 11); Credibility: Unequivocal |
| Ahmed et al. 2024 | Finding: Low levels of accepting to donate human milk were linked to (...) traditional myths; Illustration: “There are some genetic issues everything is to do with a DNA thing. Some people fear their babies to feed on the breasts of thieves, murderers, mentally disturbed people and the like that their children will end up like that." (Ahmed et al. 2024, p. 11); Credibility: Credible |
| Bressler et al. 2020 | Finding: Informally shared milk can be considered a respected and valued cultural norm; Illustration: "My milk comes from Hashem (God), my body created new milk for another baby, I am helping Hashem’s other children." (Bressler et al. 2020, p. 337); Credibility: Credible |
| Karami et al. 2025 | Finding: Many participants noted that HMB is not widely accepted in their social (...) contexts.; Illustration: “In Europe, it’s common, but here people don’t accept it easily. Without cultural acceptance and religious approval, it’s hard for people to trust it.” (Karami et al. 2025, S. 9); Credibility: Unequivocal |
| Karami et al. 2025 | Finding: Finally, participants highlighted the importance of oversight by credible health authorities (...) to enhance public trust in the system and ensure cultural and ethical alignment.; Illustration: "Without strict regulations and proper screening, I’d always be anxious about the risks.” (Karami et al. 2025, S. 7); Credibility: Unequivocal |
| Lubbe et al. 2019 | Finding: inherit some of the donor´s personlity; Illustration: “You might lose the love from mother to child.” (M3:2) “I breastfeed the child or if it is my child I give the child to someone to feed the child; there is something we call a bond when you breastfeed. So, that means that person will bond with my child.” (M3:1) (Lubbe et al. 2019, p. 5); Credibilty: Credible |
| Lubbe et al. 2019 | Finding: screening should involve testing heritage and genes; Illustration: “Some families have heritages, so I would be giving another child my heritage.” (HC3:1) “Let’s say you are a slow learner; that baby can also be a slow learner too.” (HC2:1) (Lubbe et al. 2019, p. 6); Credibilty: Credible |
| Magowan et al. 2020 | Finding: cultural belief (...) breast milk from another mother is poisonous; Illustration: “So people in our place if they hear that you’re going to express milk and give to another person, they know that you’re going to kill that person”, “that you’re going to kill the child?”, “yes, that you’re going to kill”, “so they were saying that all those who express, children die?”, “yes” FGD 4 (Magowan et al. 2020, p. 6); Credibility: Unequivocal |
| Mampane, Wolvaardt 2024 | Finding: inherite the personality traits of the donor; Illustration: /; Credibility: Not supported |

b. Wet nursing: Wet nursing that influence attitude due to donor human milk

| Ahmed et al. 2024 | Finding: Heard about wet nursing were willing to donate; Illustration: “I had my friend and her sister went abroad for greener pastures, she left a baby at 2 months and her sister used to breastfeed two babies and they grew as if they were twins and they are 3 years.. Wet nursing should be stopped completely and we resort to breast milk donation because the mother will have been tested very well at the hospital by the doctors.” (Ahmed et al. 2024, p. 8); Credibility: Unequivocal |
| --- | --- |
| Ahmed et al. 2024 | Finding: limited information; Illustration: “They will be conservative. It is something they have never heard of; they will think they are going to kill women or make them never to produce children. That kind of stuff is likely to come up as a fear from traditional leaders. That kind of fear will be put in women, and they will fear donating milk”. (R5/ FGD4, a 30-year-old mother of 3 children). (Ahmed et al. 2024, p. 11); Credibility: Unequivocal |
| Ahmed et al. 2024 | Finding: Acceptance correlated with (...) awareness about wet nursing; Illustration: “I had my friend and her sister went abroad for greener pastures, she left a baby at 2 months and her sister used to breastfeed two babies and they grew as if they were twins and they are 3 years.. Wet nursing should be stopped completely and we resort to breast milk donation because the mother will have been tested very well at the hospital by the doctors.” (Ahmed et al. 2024, p. 8); Credibility: Credible |
| Coutsoudis et al. 2011 | Finding: made the connection between wet nursing and breast milk donation; Illustration: “even in our history, we’re talking about our culture [motions to the woman next to her] if she’s still having the scanty supply, I’m having plenty, [motions to someone else], she’s having plenty, her baby cries and she’s going to toilet so I’ll just take her baby and give, and breastfeed the baby." (N2) (Coutsoudis et al. 2011, p. 3); Credibility: Unequivocal |
| Karacan et al. 2024 | Finding: consider wet nursing in case of need; Illustration: “I haven’t become a wet nurse, but I could become one for someone in need. After all, it is something that is encouraged, as the Prophet Muhammad (peace be upon him) had a wet nurse too. If I didn’t have enough breast milk, I would prefer to have a wet nurse for my baby. I would prefer someone from my close circle who I know to breastfeed my baby.” (A20). (Karacan et al. 2024, p. 5); Credibility: Unequivocal |
| Karami et al. 2025 | Finding: past experiences with breastfeeding and informal milk-sharing also influenced their perspectives; Illustration: “I’d rather give to a neighbor than a hospital bank. If I know the person, I can trust their lifestyle, their hygiene, and their religious practices. It feels more natural to share within my community than to take milk from an anonymous source.” (Karami et al. 2025, S. 8); Credibility: Unequivocal |
| Magowan et al. 2020 | Finding: seeing the practice both in hospital and also on national television; Illustration: “These days, things are not good, AIDS has come, so you can’t risk to breast feed your relative’s child because the mother has died, no, no it can’t happen.” FGD 2 (Magowan et al. 2020, p. 5); Credibility: Unequivocal |
| Coutsoudis et al. 2011 | Finding: Fear of the infant bonding with the wet nurse; Illustration: “if I breastfeed somebody’s baby that baby’s going to make a bond with me, otherwise if my baby is getting somebody else’s milk it’s going to bond with that somebody.” (M1:5) (Coutsoudis et al. 2011, p. 4); Credibility: Unequivocal |
| Karacan et al. 2024 | Finding: did not want their babies to have close physical conatct with others; Illustration:/; Credibility: Not supported |
| Lubbe et al. 2019 | Finding: fear that the infant would bond with the wet nurse; Illustration: “You might lose the love from mother to child.” (M3:2) “I breastfeed the child or if it is my child I give the child to someone to feed the child; there is something we call a bond when you breastfeed. So, that means that person will bond with my child.” (M3:1) (Lubbe et al. 2019, p. 5); Credibility: Unequivocal |
| Mampane, Wolvaardt 2024 | Finding: relationship between the recipient baby and the donor mother; Illustration: "I am asking if there will be no bond created by the intake, but if they are cleaning the milk for the concentration of germs perhaps it may address my concern on the bond that my baby can have with that donor." (P008B) (Mampane, Wolvaardt 2024, p. 5); Credibility: Unequivocal |
| Mampane, Wolvaardt 2024 | Finding: unique bond between mother and baby and the potential interefence of the use of donated milk was a real concern; Illustration: : "I will accept as long as the mother who is donating will not look at my baby in the eyes or face so that the baby will not feel or think that the person is her mother, there must not be a bond between the two." (P0015B) (Mampane, Wolvaardt 2024, p. 5); Credibility: Unequivocal |
| Mampane, Wolvaardt 2024 | Finding: raised breastfeeding benefits; Illustration : "No, I do not know why, I would like my baby to feel me, and we have a connection, it will be like drinking water, I want skin to skin and blood to blood’. There must be a bond between me and my baby. ‘My baby must feel my heartbeat." (P0023D) (Mampane, Wolvaardt 2024, p. 5); Credibility: Unequivocal |
| Ahmed et al. 2024 | Finding: limitations of wet nursing; Illustration: “I think wet nursing is not good the world has changed, and many women are sick with HIV and other diseases. It is not good it is better to donate because it is safe they test the donors and breast milk is kept safely”. (R4/FGD1, 23 years old, first-time mother). (Ahmed et al. 2024, p. 8); Credibility: Unequivocal |
| Coutsoudis et al. 2011 | Finding: wet nursing has also been deeply changed by HIV; Illustration: “a long time ago, it was right. But not now, due to these diseases. Before, there were diseases like TB but it can be cured, but the diseases today have no cure. So wet nursing today is totally wrong. It mustn’t happen now.” (M4:4) (Coutsoudis et al. 2011, p. 4); Credibility: Unequivocal |
| Lubbe et al. 2019 | Finding: no longer condoned wet nursing due to fears of diseases; Illustration: /; Credibility: not supported |
| Lubbe et al. 2019 | Finding: who had previouls practiced wet nursing were unsure whether they would do so again; They had concers about (...) HIV transmission; Illustration: “Some people don’t like to go through tests. They ask themselves, what if they are ill; those are the fears people have.” (M4:5) (Lubbe et al. 2019, p. 6) Credibility: Unequivocal |
| Magowan et al. 2020 | Finding: before era of HIV; Illustration: “These days, things are not good, AIDS has come, so you can’t risk to breast feed your relative’s child because the mother has died, no, no it can’t happen.” FGD 2 (Magowan et al. 2020, p. 5); Credibility: Unequivocal |
| Karacan et al. 2024 | Finding: be aware of her hygiene practices; Illustration: "I would still want to know the mother and be aware of her hygiene practices before making a decision” (A14). (Karacan et al. 2024, p. 6); Credibility: Unequivocal |
| Mackenzie et al. 2013 | Finding: emphasis that it was not wet nursing; Illustration: “If I was to explain to my husband what an actual milk bank is . . . that it is screened and all those sorts of things, I think he’d feel more comfortable about it.” (PSFG). (Mackenzie et al. 2013, p. 225); Credibility: credible |

2. Religion: Religious aspects that influence attitude due to donor human milk
   a. Religious values: Religious values that influence attitude due to donor human milk

| Herdy Alves et al. 2013 | Finding: Believing that this is a way for the transformation of the world; Illustration: "I question myself, why I didn’t I know and I didn’t understand the importance of donating milk. To donate is love, it is faith, it is very pleasant and it was very good for me to donate." (N7) (Herdy Alves et al. 2013, p. 5); Credibility: Credible |
| --- | --- |
| Herdy Alves et al. 2013 | Finding: imaginary myth of women; Illustration: "Donating milking is wonderful, isn´t it? It brings peace to our hearts." (N3) "I feel satisfied, I feel more human sharing my milk." (N6). "Our milk is sacred, it is made with our love." (N9). (Herdy Alves et al. 2013, p. 6); Crediblity: Credible |
| Herdy Alves et al. 2013 | Finding: Human sacredness; Illustration: "My milk is life for my baby and for the sick baby. I have a lot of milk, and that’s why I donate, it is vaccine, it is medicine." (N1) "Milk is life, it is health, I have a lot, so I can donate, once it represents life." (N10) (Herdy Alves et al. 2013, p. 6); Crediblity: Credible |
| Herdy Alves et al. 2013 | Finding: milk donation as health value, the value of the sacred and social value; Illustration: "I feel satisfied, I feel more human sharing my milk." (N6) "Our milk is sacred, it is made with our love."(N9) (Herdy Alves et al. 2013, p. 6); Crediblity: Credible |
| Bressler et al. 2020 | Finding: participants thanked God for their milk supply and the ability to feed their own infant while donating to others; Illustration: "My milk comes from Hashem (God), my body created new milk for another baby, I am helping Hashem’s other children." (Bressler et al. 2020, p. 337); Credibility: Unequivocal |
| Bressler et al. 2020 | Finding: Paricipants prayed while expressing and pumping, thinking about the recipient, their family, future, and community; Illustration: "My milk comes from Hashem (God), my body created new milk for another baby, I am helping Hashem’s other children." (Bressler et al. 2020, p. 337); Credibility: Unequivocal |
| Bressler et al. 2020 | Finding: Importance of God; Illustration: "This (my milk) is one of the most powerful things that God has created, I would routinely recite psalms over my milk before it was picked up for delivery. I thank the ‘‘man above’’ (God) who helps me to give this milk." (Bressler et al. 2020, p. 337); Credibility: Unequivocal |
| Bressler et al. 2020 | Finding: Participant understood and appreciated the magnitude their gift would have on the live of others; Illustration: "We all just help one another, we felt by doing this we were giving ourselves over to Him. It was more than actually helping the child it was providing hope for the parents and family." (Bressler et al. 2020, p. 337); Credibility: Unequivocal |
| Bressler et al. 2020 | Finding: Every child has the right to the highest available standard of health and every mother has the right to information on the benefits of human milk, both maternal and banked; Illustration: /; Credibility: Not supported |
| Faruqui et al. 2022 | Finding: feels blessed; Illustration: "…we all are sisters, we all are Adam's children… if we help each other, then Almighty Allah will also bless us with his mercy…" (FGD‐1) (Faruqui et al. 2022, p. 88); Credibility: Unequivocal |
| Karacan et al. 2024 | Finding: I would not donate; Illustration: “I definitely wouldn’t donate. It’s not a suitable practice both from a religious and health perspective. I wouldn’t give it to my child to consume either. If my milk is not sufficient or if the baby is not breast-feeding, I would give formula. It’s too risky, who knows what might happen with such a thing.” (A12) (Karacan et al. 2024, p. 5); Credibility: Unequivocal |
| Karami et al. 2025 | Finding: Some mothers hesitated due to uncertainties about the donor’s lifestyle (e.g., (...) adhered to religious dietary practices); Illustration: “I don’t know who the donor is, what they eat, if it’s halal or haram. I worry about their lifestyle, their health conditions, and whether they follow religious dietary and ethical guidelines. What if they consume something forbidden in my faith?” (Karami et al. 2025, S. 7); Credibility: Unequivocal |
| Karami et al. 2025 | Finding: While religious beliefs— particularly those related to (...) halal practices—formed the core of participants’ concerns.; Illustration: “I don’t know who the donor is, what they eat, if it’s halal or haram." (Karami et al. 2025, S. 7); Credibility: Unequivocal |
| Karami et al. 2025 | Finding: Some mothers hesitated due to (...) potential religious implications, such as whether the donor was in a state of ritual purity (ṭahārah) when expressing the milk.; Illustration: “I worry about their lifestyle, their health conditions, and whether they follow religious dietary and ethical guidelines. What if they consume something forbidden in my faith?” (Karami et al. 2025, S. 7); Credibility: Unequivocal |
| Karami et al. 2025 | Finding: Many participants noted that HMB is not widely accepted in their (...) religious contexts.; Illustration: “In Europe, it’s common, but here people don’t accept it easily. Without cultural acceptance and religious approval, it’s hard for people to trust it.” (Karami et al. 2025, S. 9); Credibility: Unequivocal |
| Loh et al. 2022 | Finding: Information for parents that DHM has been sanctioned by religious authorities; Illustration: /; Credibility: Not supported |
| Oreg, Negev 2023 | Finding: Sharing milk carries both symbolic and tangible meanings; Illustration: "Like blood, it’s something the human body produces and it doesn’t seem ethical to receive payment for it." (Oreg, Negev 2023, p. 696); Crediblity: Credible |
| Oreg, Negev 2023 | Finding: range of identities that comprise donors’ identities (Oreg, Negev 2023, p. 697); Illustration: /; Credibility: Not supported |
| Gribble 2014 | Finding: Reasons why recipient respondents hat sought peer-shared milk for their infant (Gribble 2014, p. 14); Illustration: religious obligation; Credibility: Credible |
| Oreg, Negev 2023 | Finding: Human milk is “colored” by the donor’s identity (Oreg, Negev 2023, p. 697); Illustration: /; Credibility: Not supported |
| Oreg, Negev 2023 | Finding: ask whether we can symbolically differentiate between the donor and her milk (Oreg, Negev 2023, p. 697); Illustration: /; Credibility: Not supported |
| Oueidat et al. 2020 | Finding: religious misconception that feeding donor human milk to infant is banned in the Muslim religion; Illustration: "not allowed in my religion"(#11, Oueidat et al. 2020, dataset); Credibility: Unequivocal |

b. Milk kinship: Milk kinship that influence attitude due to donor human milk

| Faruqui et al. 2022 | Finding: Religious beliefs; Illustration: "…If anyone shares, she will become that child's mother…(FGD‐1) …it's haram to marry milk siblings…" (FGD‐2) (Faruqui et al. 2022, p. 88); Credibility: Unequivocal |
| --- | --- |
| Faruqui et al. 2022 | Finding: milk siblings. This could be a reason for not sharing milk; Illustration: "In our religion, if a mother breastfeeds another child other than her own, then she becomes a milk mother of that baby. So, mostly this sharing occurs within relatives." (FGD‐3) (Faruqui et al. 2022, p. 89); Credibility: Unequivocal |
| Karacan et al. 2024 | Finding: religiously problematic; Illustration: “...From a religious perspective, there are concerns about milk siblings and wet nursing for these children. I don’t think I would take milk from these banks if I needed it. Instead, I would prefer finding a wet nurse if the need arises, God forbid. If I know who the mother is, then I might consider getting milk from these banks. " (Karacan et al. 2024, p. 4); Credibility: Unequivocal |
| Karacan et al. 2024 | Finding: views on milk sibling marriage; Illustration: “Milk brothers and sisters should not marry according to our religion. Unfortunately, it has happened too much in our country. Think about it like a brother married to a sister, oh my God, protect” (A4). (Karacan et al. 2024, p. 5); Credibility: Unequivocal |
| Karacan et al. 2024 | Finding: they did no engage in wet nursing; Illustration: “I am against everything but my own milk. I wouldn’t be a wet nurse if someone needed it. My children have never had a wet nurse. I don’t want it to happen either. I use formula even if my child has problems...” (A2). (Karacan et al. 2024, p. 5); Credibilitiy: Unequivocal |
| Karacan et al. 2024 | Finding: did not desire a wet nurse for their own baby; Illustration: “If my child needed it, I wouldn’t want her to be a wet nurse. There are formula options available...” (A26) (Karacan et al. 2024, p. 5).; Credibility: Unequivocal |
| Karacan et al. 2024 | Finding: Opinions of breast-feeding mothers on colostrum donation and usage (Karacan et al. 2024, S. 6); Illustration: “...The idea of becoming siblings can make a person uneasy. I would still approach it with suspicion. After all, there will definitely be my own colostrum. No, I would only give my own colostrum.” (A1) (Karacan et al. 2024, p. 5); Credibility: Unequivocal |
| Karami et al. 2025 | Finding: gender of the donor’s child as a critical factor; Illustration: "If the donor’s child is the same gender as mine, I’d be even more careful”, “Boys or girls of the same age might grow close later in life, so I need to make sure we know exactly who their milk siblings are to prevent any future issues. It’s not just about health — it’s about protecting them socially and religiously too." (Karami et al. 2025, S. 8); Credibility: Unequivocal |
| Karami et al. 2025 | Finding: While religious beliefs— particularly those related to milk kinship, (...) —formed the core of participants’ concerns.; Illustration: “Milk makes siblings; I don’t want my child tied to a stranger. What if, in the future, my child unknowingly meets and forms a relationship with someone who is their milk sibling? Without clear records or transparency, how can we ensure that such relation (...)." (Karami et al. 2025, S. 7); Credibility: Unequivocal |
| Karami et al. 2025 | Finding: need for clear documentation and transpareny to adress these concerns; Illustration: "Without clear records or transparency, how can we ensure that such relationships don’t happen? It worries me because, in our faith, milk kinship is just as significant as blood ties.” (Karami et al. 2025, S. 7 - 8); Credibility: Unequivocal |
| Kürtüncü et al. 2024 | Finding: The mothers stated that donating breast milk would establish a familial relationship known as 'milk kinship,' (...). They expressed disapproval of human milk banks (...).; Illustration: “If I got milk from milk banks, I wouldn’t know who I’m getting it from, I wouldn’t know the gender of the woman’s child. This would be a problem when my child grows up. It is forbidden in my religion.” (M11). (Kürtüncü et al. 2024, S. 4); Credibility: Unequivocal |
| Loh et al. 2022 | Finding: Muslim participants expressed awarness of milk kinship; Illustration: “In our faith, once you receive donor´s milk from a different mother, that person is considered the second mother (M6) (Loh et al. 2022, p. 352); Credibility: Unequivocal |
| Mondkar et al. 2018 | Finding: refusal to donate because of religious reasons, Illustration: "There will be an obligation lifelong if we take someone else’s milk.’’ (Mondkar et al. 2018, p. 698), Credibility: Unequivocal |
| Mondkar et al. 2018 | Finding: Life-long obligation; Illustration: ‘‘There will be an obligation lifelong if we take someone else’s milk.’’ (Mondkar et al. 2018, p. 698), Credibility: Unequivocal |
| Oueidat et al. 2020 | Finding: opposing the idea because of religious beliefs; Illustration: "not permitted in my religion", "˝Religiously, the other woman would be his mother" (#103, Oueidat et al. 2020, dataset); Credibility: Unequivocal |

c. Lack of religious knowledge: Lack of knowledge regarding religious obligations according donor human milk

| Faruqui et al. 2022 | Finding: concerned about the religious restrictions; Illustration: "According to Islamic Shariah [the correct path that Muslims follow to live moral lives], it is haram to marry milk siblings. This could be a reason for not sharing milk." (FGD‐2) (Faruqui et al. 2022, p. 89); Credibility: Unequivocal |
| --- | --- |
| Gelano et al. 2018 | Finding: concerned that human breast milk sharing is prohibited; Illustration: "rather it is better to feed formula or cow milk by bottle. In the Muslim religion we do not support feeding someone else’s breast milk to our infants. Because it is prohibited.” (Gelano et al. 2018, p. 6); Credibility: Unequivocal |
| Karacan et al. 2024 | Finding: By providing accurate religious guidance, mothers can feel more confident in using; Illustration: “I haven’t heard anything like that in the Quran, but if it’s mentioned, I would consider donating colostrum as it would be beneficial for health. " (Karacan et al. 2024, p. 5); Credibility: Unequivocal |
| Karami et al. 2025 | Finding: Endorsement from trusted authorities, particularly healthcare professionals and religious scholars, significantly influenced attitudes toward HMB; Illustration: “If a doctor said it’s safe and good, I’d trust it more. If a paediatrician or religious scholar endorsed it and explained the safety measures, I’d feel more comfortable.” (Karami et al. 2025, S. 9); Credibility: Unequivocal |
| Karami et al. 2025 | Finding: Addressing cultural concerns through religious discourse, community discussions, and awareness programs may facilitate greater acceptance of HMB within conservative societies.; Illustration: “In Europe, it’s common, but here people don’t accept it easily. Without cultural acceptance and religious approval, it’s hard for people to trust it.” (Karami et al. 2025, S. 9); Credibility: Credible |
| Loh et al. 2022 | Finding: struggled between their baby´s needs and the perceived religious implications of recieving DHM; Illustration: “My husband… was asking me… if a Chinese mother give [donates] milk right, maybe the Chinese mother… eats… pork or something like that… so… halal or non halal.” (M5) (Loh et al. 2022, p. 352), Credibility: Unequivocal |
| Loh et al. 2022 | Finding: were unsure of its exact criteria and concluded that milk kinship would not apply to them due to donor anonymity; Illustration: /; Credibility: not supported |

3. Dyad composition: Claims according participants composition because of religious or cultural values
   a. General aspects regarding dyad composition: General aspects according participants composition because of religious or cultural values

| Oreg, Negev 2023 | Finding: peer-to-peer milk-sharing enables a better personal match between donor and recipient preferences; Illustration: /; Credibility: Not supported |
| --- | --- |
| Coutsoudis et al. 2011 | Finding: Donor´s identity is relevant; Illustration: “my other fear is that women talk amongst themselves, and if you tell someone that your baby’s getting donated breast milk and later when the baby’s older they tell him, he’ll want to know what that mother was.” (M1:1) (Coutsoudis et al. 2011, S. p); Credibility: Unequivocal |
| Gribble 2013 | Finidng: Prefer to know the recipient; Illustration: “I think that would be too impersonal for me. I like knowing who is getting my breastmilk.” (Gribble 2013, p. 455); Credibility: Unequivocal |
| Gribble 2013 | Finding: to take account of the relationsship created by milk sharing; Illustration: "to take account of the relationship created by the sharing of milk" (Gribble 2013, p. 456); Credibility: Unequivocal |
| Karacan et al. 2024 | Finding: Muslim families would like to know each other is to prevent milk sibling marriages; Illustration: "...I know they can’t get married. Since I do not know from whom I give milk and from whom I receive milk, children will ot be able to know that they are siblings. This situation affects me a lot from a religious point of view.” (A18) (Karacan et al. 2024, p. 5); Credibility: Credible |
| Karami et al. 2025 | Finding: While religious beliefs— particularly those related to (...) lineage, (...) —formed the core of participants’ concerns.; Illustration: “If I knew the donor was a good, religious person, I’d use it. I need to be sure that they follow halal practices, have good moral character, and live a healthy lifestyle." (Karami et al. 2025, S. 8); Credibility: Unequivocal |
| Karami et al. 2025 | Finding: Some mothers hesitated due to uncertainties about the donor’s lifestyle (e.g., (...) adhered to religious dietary practices); Illustration: “I don’t know who the donor is, what they eat, if it’s halal or haram. I worry about their lifestyle, their health conditions, and whether they follow religious dietary and ethical guidelines. What if they consume something forbidden in my faith?” (Karami et al. 2025, S. 7); Credibility: Unequivocal |
| Karami et al. 2025 | Finding: Many mothers preferred milk from known and trusted sources rather than relying on formal milk banks.; Illustration: “If I knew the donor was a good, religious person, I’d use it." (Karami et al. 2025, S. 8); Credibility: Unequivocal |
| Karami et al. 2025 | Finding: gender of the donor’s child as a critical factor; Illustration: "If the donor’s child is the same gender as mine, I’d be even more careful”, “Boys or girls of the same age might grow close later in life, so I need to make sure we know exactly who their milk siblings are to prevent any future issues. It’s not just about health — it’s about protecting them socially and religiously too." (Karami et al. 2025, S. 8); Credibility: Unequivocal |
| Karami et al. 2025 | Finding: need for clear documentation and transpareny to adress these concerns; Illustration: "Without clear records or transparency, how can we ensure that such relationships don’t happen? It worries me because, in our faith, milk kinship is just as significant as blood ties.” (Karami et al. 2025, S. 7 - 8); Credibility: Unequivocal |
| Karami et al. 2025 | Finding: While religious beliefs— particularly those related to (...) halal practices—formed the core of participants’ concerns.; Illustration: “I don’t know who the donor is, what they eat, if it’s halal or haram." (Karami et al. 2025, S. 7); Credibility: Unequivocal |
| Karami et al. 2025 | Finding: Some mothers hesitated due to (...) potential religious implications, such as whether the donor was in a state of ritual purity (ṭahārah) when expressing the milk.; Illustration: “I worry about their lifestyle, their health conditions, and whether they follow religious dietary and ethical guidelines. What if they consume something forbidden in my faith?” (Karami et al. 2025, S. 7); Credibility: Unequivocal |
| Kürtüncü et al. 2024 | Finding: Some mothers mentioned relying on breast milk banks after the regular registration system was established for milk collection. This allowed the brothers to become acquainted with each other.; Illustration: "It is very inconvenient for me to give someone else's milk to my child. It is equally a problem that I give my own milk to the president's child. However, I would only do it if I knew the person."(Kürtüncü et al. 2024, S. 5); Credibility: Unequivocal |
| Mampane, Wolvaardt 2024 | Finding: Participants were unaware of this programme an drefelcted on the cultural aspect, Illustration: "It is an emotional process from a cultural point of view." (Mampane, Wolvaardt 2024, p. 5), Credibility: Unequivocal |
| Coutsoudis et al. 2011 | Finding: Related the question to blood donation, saying the race does not matter; Illustration: “even the blood is the same. It doesn’t depend on skin color.” (G1) (Coutsoudis et al. 2011, p. 7); Credibility: Unequivocal |
| Rabinowitz et al. 2018 | Finding: knowing the milk provider pesonally would make them more mistrustful; Illustration: ‘‘I don’t know exactly their medical background and [they haven’t] been screened’’ (#16, would choose HDM) (Rabinowitz et al. 2018, p. 201); Credibility: Unequivocal |

b. Preference for family: Preference of sharing between family because of religious or cultural values

| Chugh Sachdeva et al. 2019 | Finding: wet nursing was an age old and common practice - were comfortable with it if practiced within the family; Illustration: /; Credibility: Not supported |
| --- | --- |
| Faruqui et al. 2022 | Finding: Preferably from close family members; Illustration: "In fact, we can breastfeed our babies by our relatives or family members." (FGD‐2) (Faruqui et al. 2022, p. 85); Credibility: Unequivocal |
| Gelano et al. 2018 | Finding: only be possible if recipient´s baby has a blood relationship with the donor; Illustration: “I do not understand about human breast milk banking. In the past I have heard information about wet nurses but this is only possible if we have a blood relation with the baby’s family. For example, my sister can feed my baby if I cannot feed my baby otherwise it is not possible.” (Gelano et al. 2018, p. 6); Credibility: Unequivocal |
| Gelano et al. 2018 | Finding: religious reservations; Illustration: “I do not understand about human breast milk banking. In the past I have heard information about wet nurses but this is only possible if we have a blood relation with the baby’s family. For example, my sister can feed my baby if I cannot feed my baby otherwise it is not possible.” (Gelano et al. 2018, p. 6); Credibility: Unequivocal |
| Lubbe et al. 2019 | Finding: Willing to practice wet nursing if they knew the other person involved, and knew her status; Illustration: /; Credibility: not supported |
| O´Sullivan et al. 2016 | Finding: altruistic motivation; Illustration: "My sister is currently pregnant and if I knew that she was having problems with nursing or couldn’t nurse for some reason, like, I would definitely hand it right over to her." (Sadie, nonsharer) (O´Sullivan et al. 2016, p. 419); Credibility: Unequivocal |
| O´Sullivan et al. 2016 | Finding: Sharing occured between family; Illustration: ". . . my sister-in-law. . . . I was giving her some frozen milk for a little while. I forgot about that." (Nora, provider) (O´Sullivan et al. 2016, p. 420); Credibility: Unequivocal |

c. Preference for friends: Preference of sharing between friends because of religious or cultural values

| O´Sullivan et al. 2016 | Finding: Sharing occured between (...) friends; Illustration: "[My friend] called me and asked if she could have this, all the milk that I had stockpiled, and so I gave her like hundreds of ounces of milk." (Olive, provider) (O´Sullivan et al. 2016, p. 420); Credibility: Unequivocal |
| --- | --- |
| Rabinowitz et al. 2018 | Finding: mixed attitude using a friend; Illustration: ‘‘in foreign countries, if the mom died at birth. and there was somebody in their tribe. who was already breastfeeding. they’d just jump in and take care, and I think that’s beautiful’’ (#3, would choose HDM). Rabinowitz et al. 2018, p. 201), Credibility: Unequivocal |

d. Preference for own community: Preference of sharing between community because of religious or cultural values

| Bressler et al. 2020 | Finding: Social Connectedness; Illustration: "It was very easy to do because all the instructions are online, I learned how to make my body make more milk, so I still had enough for my baby at home. The women were all supportive and encouraging online it was like one big family working together to care for our community." (Bressler et al. 2020, p. 338); Credibility: Unequivocal |
| --- | --- |
| Bressler et al. 2020 | Finding: connected with several other mothers in the Orthodox community; Illustration: "I got a message from a woman who said she was looking to find out if I still had milk available because there was a sick baby in need so I wanted to help more so I called all of the nursing mothers I personally knew and five of them donated to this child." (Bressler et al. 2020, p. 338); Credibility: Unequivocal |
| Bressler et al. 2020 | Finding: That they were another Orthodox family in need and that was all they needed to know; Illustration: "We all just help each other because we can. It doesn’t make any difference who it is going to so long as the baby needs it, we do it. Five of us pulled together to help save the baby, we never got to see him but knew it would help him, so we all worked together and the five of us fed that baby for several weeks." (Bressler et al. 2020, p. 339); Credibility: Unequivocal |
| Bressler et al. 2020 | Finding: would help any baby in their community in need; Ilustration: "I would do anything for any baby in my community, most of us felt that way so we just do it, we feel that if we could we always should. I guess, you know we just help each other, we give our all." (Bressler et al. 2020, p. 339); Crediblity: Unequivocal |
| Bressler et al. 2020 | Finding: prefer to share within their community first; Ilustration: "I would do anything for any baby in my community, most of us felt that way so we just do it, we feel that if we could we always should. I guess, you know we just help each other, we give our all." (Bressler et al. 2020, p. 339); Crediblity: Unequivocal |
| Bressler et al. 2020 | Finding: Value because of the fact that mothers adhered to a kosher diet; Illustration: "I would do anything for any baby in my community, most of us felt that way so we just do it, we feel that if we could we always should. I guess, you know we just help each other, we give our all." (Bressler et al. 2020, p. 339); Crediblity: Credibile |
| Bressler et al. 2020 | Finding: Milk would be considered Kosher and this is not something that would available to families from nonprofit milk banks; Illustration: "I would do anything for any baby in my community, most of us felt that way so we just do it, we feel that if we could we always should. I guess, you know we just help each other, we give our all." (Bressler et al. 2020, p. 339); Crediblity: Credible |
| Bressler et al. 2020 | Finding: emotional and social value placed on informal donation; Illustration: "I would do anything for any baby in my community, most of us felt that way so we just do it, we feel that if we could we always should." (Bressler et al. 2020, p. 339); Crediblity: Credible |
| Faruqui et al. 2022 | Finding: Hoping some else does the same if needed; Illustration: "Once my neighbour went to the hospital and the baby was left at home and was crying for milk…the family members were trying to feed formula milk but the baby was not drinking it. So I breastfed and calmed down the baby. I believe that, if I help others, then others will do the same for me." (FGD‐1) (Faruqui et al. 2022, p. 88); Credibility: Unequivoal |
| Gribble 2013 | Finding: must know her donors to determine that they were also Muslim; Illustration: “The mothers must be Muslim and is breastfeeding their children as well . . . frequent visit to the milk donor is important to keep track on their location.” (Gribble 2013, p. 457); Credibility: Unequivocal |
| Lubbe et al. 2019 | Finding: testing (...) genes to ensure that the cultures were compatible; Illustration: “Some families have heritages, so I would be giving another child my heritage.” (HC3:1) “Let’s say you are a slow learner; that baby can also be a slow learner too.” (HC2:1) (Lubbe et al. 2019, p. 6); Credibilty: Credible |
| O´Sullivan et al. 2016 | Finding: informal HM sharing is likely occuring at the community; Illustration: "A couple of weeks or month ago . . . [my husband] heard a story about a mother who had died during giving birth and . . . that there might come up a call for donating milk. " (Freya, nonsharer) (O´Sullivan et al. 2016, p. 419); Credibility: Credible |
| Oreg, Negev 2023 | Finding: whether they are practicing Jewish law or not, symbolically affect the “quality” of their milk (Oreg, Negev 2023, p. 697); Illustration: /; Credibility: Not supported |
| Vesel et al. 2023 | Finding: Concerns about dismatch of (...) religious backgrounds; Illustration:/, Credibility: Credible: Not supported |

4. Network: Perspective and education of parents´ network towards DHM
   a. General aspects regarding families´ network: General aspects according the role of community due to attitude due to donor human milk

| Esquerra-Zwiers et al. 2016 | Finding: the early consent process (...) did not permit discussion of DHM with family, friends, or religious leaders; Illustration: "And before I said okay, I actually had to talk to a few of my friends who pretty much all had premature babies." (Esquerra-Zwiers et al. 2016, p. 6); Credibility: Credible |
| --- | --- |
| Karacan et al. 2024 | Finding: pressure from (...) community; Illustration: “...From a religious perspective, there are concerns about milk siblings and wet nursing for these children. I don’t think I would take milk from these banks if I needed it. Instead, I would prefer finding a wet nurse if the need arises, God forbid." (Karacan et al. 2024, p. 4); Credibility: Credible |
| Karami et al. 2025 | Finding: Many participants experienced profound shame and a sense of failure when unable to breastfeed.; Illustration: "If I rely on HMB, I worry that I’m failing my child or that others will judge me for not being ‘enough’ as a mother." (Karami et al. 2025, S. 6); Credibility: Unequivocal |
| Mackenzie et al. 2013 | Finding: they felt they need not tell anyone who they might be unsupportive; Illustration: “A lot of the people I talk to about breastfeeding . . . seem to have a very positive view. Either way, if I was a donor or a recipient I think that people would be positive.” (Abby BF) (Mackenzie et al. 2013, p. 225); Credibility: Credible |

b. Influence by family: Role of family according attitude due to donor human milk

| Ahmed et al. 2024 | Finding: Role of her grandmother; Illustration: “I think the community leaders will buy the idea because traditional leaders know less of formulas, so they know that breastfeeding is on point for babies. I had a premature baby at 7 months, and I had no milk. They called my granny and they told her about it she told them that they should call my aunt and she gets tested and she helps me to get breast milk for my baby”. (Ahmed et al. 2024, p. 12); Credibility: Unequivocal |
| --- | --- |
| Herdy Alves et al. 2013 | Finding: limits as to the comprehension of the importance of donation; Illustration: "I have lot of information at home, my whole family says that the milk must be only for my baby, once donating milk can make my milk weak and it will be over." (N2) (Herdy Alves et al. 2013, p. 5); Credibility: Credible |
| Bressler et al. 2020 | Finding: sensitivity and incorporation of the families’ core values, which are often influenced by their culture and/or faith traditions; Illustration: "My milk comes from Hashem (God), my body created new milk for another baby, I am helping Hashem’s other children." (Bressler et al. 2020, p. 337); Credibility: Credible |
| Faruqui et al. 2022 | Finding: need of obtaining approval from their family members; Illustration: "It is a must to take permission from the husband before breastmilk sharing." (FGD‐1) (Faruqui et al. 2022, p. 87); Credibility: Unequivocal |
| Faruqui et al. 2022 | Findings: to avoid social repercussions; Illustration: "Some families tell their daughter‐in‐law not to share their breast milk with others, if they do then they will not tolerate this… what should that mother do then? It's better for her not to share because ultimately she has to live with her family and follow their rules and regulations." (FGD‐1) (Faruqui et al. 2022, p. 89); Credibility: Unequivocal |
| Karacan et al. 2024 | Finding: Pressure from (...) family; Illustration: "My family wouldn’t want it, and neither would I or my spouse." (Karacan et al. 2024, p. 5); Credibility: Unequivocal |
| Karami et al. 2025 | Finding: This sentiment illustrates how (...) familital pressures intensified mothers’ emotional burdens, heightening fears of inadequacy associated with using HMB.; Illustration: "If I rely on HMB, I worry that I’m failing my child or that others will judge me for not being ‘enough’ as a mother." (Karami et al. 2025, S. 6); Credibility: Unequivocal |
| Karami et al. 2025 | Finding: hierarchical nature of family decision-making, where societal and familial pressures can limit a mother’s autonomy; Illustration: “My mom says it’s not clean, my husband decides what’s best. In our family, elders have a big say in how we raise our children, and if they don’t trust milk banks, it’s hard for me to go against them.” (Karami et al. 2025, S. 8); Credibility: Unequivocal |
| Mackenzie et al. 2013 | Finding: Consistent information and support, particulary from family (...) is essential; Illustration: "We have a milk bank,” and took it from there, because they’re your main contacts during that period." (Cecilia PS) (Mackenzie et al. 2013, p. 227); Credibility: Credible |
| Magowan et al. 2020 | Finding: Influenced by family; Illustration: “If he refuses, then I have to leave it (using DHM) because they can chase me away from the home.” FGD 6 (Magowan et al. 2020, p. 6); Credibility: Credible |
| Vesel et al. 2023 | Finding: Lack of support from family and community; Illustration: /, Credibility: not supported |

c. Influence by friends: Role of friends according attitude due to donor human milk

| Mackenzie et al. 2013 | Finding: friends were less a constraint; Illustration: “A lot of the people I talk to about breastfeeding . . . seem to have a very positive view. Either way, if I was a donor or a recipient I think that people would be positive.” (Abby BF) (Mackenzie et al. 2013, p. 225); Credibility: Credible |
| --- | --- |
| Mackenzie et al. 2013 | Finding: Consistent information and support, particulary from (...) friends (...) is essential; Illustration: "We have a milk bank,” and took it from there, because they’re your main contacts during that period." (Cecilia PS) (Mackenzie et al. 2013, p. 227); Credibility: Credible |

d. Influence by husband: Role of husbands according attitude due to donor human milk

| Ahmed et al. 2024 | Finding: men preferred to have women with firm breasts; Illustration: “Our spouses also don’t like floppy boobs (breasts) they keep looking for young shooting boobs. That is why women breastfeed for a short while and then put them on the formula due to the demands of men”. (R7/FGD1, 23 year old, a mother of 2 children). (Ahmed et al. 2024, p. 10); Crediblity: Unequivocal |
| --- | --- |
| Ahmed et al. 2024 | Finding: Souse refusal; Illustration: “One time my hubby asked me, don’t you think you are depleting for our baby. And I would tell him let us share. He was hesitant first but later had to allow me. But then another friend of mine the husband refused her to give his son’s milk”. (Mother of one, and a frequent donor at Nsambya Hospital). (Ahmed et al. 2024, p. 11); Crediblity: Unequivocal |
| Ahmed et al. 2024 | Finding: Spouses were supportive; Illustration: "My husband was good and supported me in donating breast milk to those who were in need of it”. (KII4, frequent breast milk donor at Nsambya Hospital). (Ahmed et al. 2024, p. 11); Crediblity: Unequivocal |
| Ahmed et al. 2024 | Finding: male partner engagement in human milk donation could further increase the acceptablity; Illustration: “Mine was good and supported me, but others refuse their wives to donate their babies’ milk; it will be reduced, and it is not enough for their baby”. (KII4, frequent donor and staff at Nsambya Hospital). (Ahmed et al. 2024, p. 11); Cedibility: Unequivocal |
| Faruqui et al. 2022 | Finding: permission from the husband; Illustration: "It is a must to take permission from the husband before breastmilk sharing." (FGD‐1) (Faruqui et al. 2022, p. 5); Credibility: Unequivocal |
| Faruqui et al. 2022 | Finding: ´Sinful act´ if the husband´s permission was not taken; Illustration: "If the husband permits, then it's okay to share. Otherwise, it would be a sinful act." (FGD‐4) (Faruqui et al. 2022, p. 88); Credbility: Unequivocal |
| Karacan et al. 2024 | Finding: Pressure from spouses; Illustration: "My family wouldn’t want it, and neither would I or my spouse." (Karacan et al. 2024, p. 5); Credibility: Credible |
| Mackenzie et al. 2013 | Finding: Support from their partner (all were partnered) was crucial; Illustration: “A lot of the people I talk to about breastfeeding . . . seem to have a very positive view. Either way, if I was a donor or a recipient I think that people would be positive.” (Abby BF) (Mackenzie et al. 2013, p. 225); Crediblity: Credible |

e. Influence by traditional/religious leaders: Role of community/religious leaders according attitude due to donor human milk

| Ahmed et al. 2024 | Finding: community leaders/elders were not conversant with formula; Illustration: “Some might be young and might not understand these issues well, but traditional leaders I think will encourage us to donate some breast milk. They know the value of breast milk and always encourage us to breastfeed our babies longer. So I feel what they need is sensitization and have information about donation. They will support the idea”. (R8/FGD1, 23 years old, a mother of 5 children)(Ahmed et al. 2024, p. 12); Credibily: Unequivocal |
| --- | --- |
| Ahmed et al. 2024 | Finding: community leaders and elderely people would facilitate; Illustration: ".. we need to train the traditional and community leaders about breast milk donation. What they know most of is wet nursing, so we need to sensitize them and de-campaign that exercise of another mother breastfeeding another’s baby and promote donation of breast milk.” (R4/FGD1, 23-year-old first-time mother). (Ahmed et al. 2024, p. 12); Credibility: Unequivocal |
| Karami et al. 2025 | Finding: Finally, participants highlighted the importance of oversight by credible health authorities and/or respected religious leaders (...).; Illustration: "Without strict regulations and proper screening, I’d always be anxious about the risks.” (Karami et al. 2025, S. 7); Credibility: Unequivocal |
| Lubbe et al. 2019 | Finding: When asked about perceptions of community leaders and traditional healers (...), the participants had mixed responses; Illustration: /; Credibilty: Not supported |
| Lubbe et al. 2019 | Finding: community leaders and traditional leaders should also be involved as they influence cummunities´ decisions and perception; Illustration:/; Credibility: Not supported |
| Magowan et al. 2020 | Finding: religious leaders and community elders (...) did not have relevant knowledge; Illustration: “So people in our place if they hear that you’re going to express milk and give to another person, they know that you’re going to kill that person”, “that you’re going to kill the child?”, “yes, that you’re going to kill”, “so they were saying that all those who express, children die?”, “yes” FGD 4 (Magowan et al. 2020, p. 6); Credibility: Unequivocal |
| Magowan et al. 2020 | Finding: religious leaders and community elders (...) not (...) influential; Illustration: “So people in our place if they hear that you’re going to express milk and give to another person, they know that you’re going to kill that person”, “that you’re going to kill the child?”, “yes, that you’re going to kill”, “so they were saying that all those who express, children die?”, “yes” FGD 4 (Magowan et al. 2020, p. 6); Credibility: Credible |

f. Influence by health care worker: Role of health care workers according attitude due to donor human milk

| Karami et al. 2025 | Finding: Finally, participants highlighted the importance of oversight by credible health authorities and/or respected religious leaders (...).; Illustration: "Without strict regulations and proper screening, I’d always be anxious about the risks.” (Karami et al. 2025, S. 7); Credibility: Unequivocal |
| --- | --- |
| Mackenzie et al. 2013 | Finding: Consistent information and support, particulary from (...) health care workers is essential; Illustration: "We have a milk bank,” and took it from there, because they’re your main contacts during that period." (Cecilia PS) (Mackenzie et al. 2013, p. 227); Credibility: Credible |
| Magowan et al. 2020 | Finding: Power of (...) HCWs; Illustration: "I need first to ask my husband, if he accepts then I accept.” FGD 3 (Magowan et al. 2020, p. 6); Credibility: Credible |

g. Need of education: Lack of knowledge of parents´ network

| Ahmed et al. 2024 | Finding: Community education; Illustration: “I think ladies should be taught about it and they will be willing to donate it is about awareness creation. They need to know that some mothers lack breast milk or that some preterm infants have lost their mothers. When they learn and see what happens in the NICU, then they will be kind to donate”. (R6/FGD2, 25-year-old mother of 3 children). (Ahmed et al. 2024, p. 12); Credibility: Unequivocal |
| --- | --- |
| Ahmed et al. 2024 | Finding: community education (...) could further increase the acceptabilty; Illustration: “Awareness creation is very important to these ladies now like my other baby I had a lot of breast milk, and I would just waste it. I didn’t know that some people look for breast milk for their preterm when they don’t have. I could leave it to pour so that it reduces had I know I would have come and donate”. (R5/FGD4, a 30-year-old mother of 3 children). (Ahmed et al. 2024, p. 12); Credibility: Credible |
| Bressler et al. 2020 | Finding: Understanding these cultural practices and working with this community can lead to a more satisfying breastfeeding experience among Ultra-Orthodox Orthodox Jewish families; Illustration:/; Credibility: Not supported |
| Chugh Sachdeva et al. 2019 | Finding: Important to raise awareness among them on its harms and healthier alternatives; Illustration:/; Credibility: Not supported |
| Coutsoudis et al. 2011 | Finding: education should be extendet to family members and broader community; Illustration: “if you can teach [the mothers], they’re the ones who are going to tell us at home. But we need to be taught, but we don’t have time.” (P3) (Coutsoudis et al. 2011, p. 8); Credibility: Unequivocal |
| Coutsoudis et al. 2011 | Finding: importance of educating grandmothers; Illustration: “the grannies need to be told what is happening these days. In the past there were no diseases and so [behaviors were different], and now they’re getting information from all sorts of different people. And now the grannies are scared about these diseases. Even if I’m not at home and I express my breast milk, she’ll be more understanding because she’ll have heard this information.” (M2:5) (Coutsoudis et al. 2011, p. 8); Credibility:Unequivocal |
| Coutsoudis et al. 2011 | Finding: none of the participants offered specific ideas on ways of reaching family members; Illustration: “yes, if it’s advocated not just in our setting but through government and media. It’s actually fashionable.” (D2) (Coutsoudis et al. 2011, p. 8); Credibility: Unequivocal |
| Gelano et al. 2018 | Finding: Did not know; Illustration: “I have heard about donated human milk for infants feeding from other countries, such kinds of practices are especially present in orphanages but in our country there are no such kinds of practice.” (Gelano et al. 2018, p. 6); Credibility: Unequivocal |
| Karacan et al. 2024 | Finding: beneficial to provide information about milk banks to the public and mothers and to share positive views from religious authorities wirh the community; Illustration: “...From a religious perspective, there are concerns about milk siblings and wet nursing for these children. I don’t think I would take milk from these banks if I needed it. Instead, I would prefer finding a wet nurse if the need arises, God forbid." (Karacan et al. 2024, p. 4); Credibility: Credible |
| Karami et al. 2025 | Finding: Public awareness campaigns, featuring testimonials from respected medical professionals and religious leaders, could help alleviate concerns and improve acceptance rates.; Illustration:“If a doctor said it’s safe and good, I’d trust it more. If a paediatrician or religious scholar endorsed it and explained the safety measures, I’d feel more comfortable.” (Karami et al. 2025, S. 9); Credibility: Unequivocal |
| Loh et al. 2022 | Finding: healthcare practitioners should approach mothers in a culturally sensitive manner; Illustration: /; Credibility: Not supported |
| Lubbe et al. 2019 | Finding: education should also include grandmothers; Illustration:/; Credibility: Not supported |
| Mondkar et al. 2018 | Finding: Dissemination of information on milk donation in the community; Illustration: ‘‘There will be an obligation lifelong if we take someone else’s milk.’’ (Mondkar et al. 2018, p. 698); Credibility: Unequivocal |
