# Supplementary material for: Parents' Transcultural Attitudes and Knowledge Concerning Human Milk Exchange: A Qualitative Systematic Review and Meta‐Aggregation
Source: Matern Child Nutr. 2026 Oct 27;22(4):e70278. doi: 10.1111/mcn.70278 (PMC13714566; doi:10.1111/mcn.70278)
Supplement: Supplementary file 3 [file MCN-22-e70278-s003.docx]

Supplementary material 3: Data extraction I

| Author | Title | Year | DOI | Funding | Conflict of interest | Data collection | Data analysis | Sampling | Sample Size | Phenomena of interst | Interviewvee | Setting | Country of origin | Patients | Type of HME | Religion |
| --- | --- | --- | --- | --- | --- | --- | --- | --- | --- | --- | --- | --- | --- | --- | --- | --- |
| Mohammed A. M. Ahmed, Charles Patrick Namisi, Nakibuuka Victoria Kirabira, Micheal Webba Lwetabe and Joseph Rujumba | Acceptability to donate human milk among postnatal mothers at St. Francis hospital Nsambya, Uganda: a mixed method study | 2024 | https://doi.org/10.1186/s13006-024-00615-2 | Funded by the Ahmed M. and supported by Mother Kevin Post Graduate Medical School, Uganda Martyrs University | None declared. | focus group discussions | Content thematic approach (Graneheim, Lundman 2004) | providing information to postnatal mothers who were attending the postnatal clinic and department of paediatrics at St. Francis Hospital Nsambya | 30 | Acceptability to donate human milk | mothers | Hospital | Uganda | unknown | Unknown/mixed | unknown |
| Valdecyr Herdy Alves, Diego Pereira Rodrigues, Maria Bertilla Lutterbach Riker Branco, Rosangela de Matos Pereira de Souza, Renata Rangel Birindiba de Souza, Flávia do Valle Andrade Medeiros | HUMAN MILK BANK UNDER THE PERSPECTIVE OF THE DONATING WOMAN | 2013 | <https://doi.org/10.15253/2175-6783.20130006000013> | None declared. | None declared. | individual  interviews | Formulation of thematic category (Minayo 2010) | Recruiting through Human Milk Bank | 11 | values related to the act of milk donation | women/ nursing mother | Hospital | Brazilian territory | unknown | formal | unknown |
| Toby Bressler, Laura M. Dambra-Candelaria and Diane L. Spatz | Informal Milk Sharing for the Hospitalized at-Risk Infant in the Ultra-Orthodox-Haredi Jewish Community in the United States | 2020 | DOI: 10.1089/bfm.2019.0216 | Supported by a faculty grant from the Molloy College Hagan School of Nursing | None declared. | semistructured telephone interviews | Edmund Husserl’s philosophical framework and Colaizzi’s method of analysis | mothers were recruited through snowball technique, personal networks, and social media connections | 14 | experience of informal donation | Mothers | Community | Northeastern region of the United States | unknown | Informal | Orthodox Jewish |
| Ruchika Chugh Sachdeva, Jayashree Mondkar, Sunita Shanbhag, Minu Manuhar, Aisha Khan, Rajib Dasgupta, and Kiersten Israel-Ballard | A Qualitative Analysis of the Barriers and Facilitators for Breastfeeding and Kangaroo Mother Care Among Service Providers, Mothers and Influencers of Neonates Admitted in Two Urban Hospitals in India | 2019 | DOI: 10.1089/bfm.2018.0177 | None declared. | None declared. | in-depth interviews | Thematic analysis | None declared. | 56 | Barriers and Facilitators for Breastfeeding and Kangaroo Mother Care | service providers, mothers, fathers, grandmothers | Hospital | Mumba | unknown | Unknown/mixed | unknown |
| Irene Coutsoudis, Alissa Petrites, Anna Coutsoudis | Acceptability of donated breast milk in a resource limited South African setting | 2011 | doi:10.1186/1746-4358-6-3 | Supported by Carl and Emily Fuchs Foundation | None declared. | focus group discussions | Qualitative content analysis (Graneheim and Lundman and Ulin and colleagues) | selected either through patient records of a local clinic and its staff | 48 | attitudes towards donated breast milk | mothers male partners, grandmothers | Hospital | South Africa | unknown | Unknown/mixed | unknown |
| Anita Esquerra-Zwiers, Beverly Rossman, Paula Meier, Janet Engstrom, Judy Janes and Aloka Patel | “It’s Somebody Else’s Milk”: Unraveling the Tension in Mothers of Preterm Infants Who Provide Consent for Pasteurized Donor Human Milk | 2016 | doi:10.1177/0890334415617939. | Funded by NIH grants: NR010009 and NICHD R03HD081412. | None declared. | in-depth semistructured interviews | Content analysis (Hsieh HF, Shannon SE 2005) | Initial convenience and purposive sampling | 20 | maternal decision-making process in providing consent for DHM feedings. | mothers | unclear | Chicago | premature infants | formal | unknown |
| Wafi A. Faruqui, Fahima Hossain, Shahrin E. Rayna, Khan Mohammad Thouhidur Rahman, Md. Salman M. Piyal, Md. Khalequzzaman | Perceived facilitators and barriers towards acceptability of human milk sharing among mothers in Dhaka, Bangladesh | 2023 | DOI: 10.1002/rfc2.37 | None declared. | None declared. | focus group discussions | Content analysis (Hsieh HF, Shannon SE 2005) | Identification while attending the Expanded Program on Immunisation (EPI) centres | 279 | facilitators and barriers towards participating in or rejecting human milk sharing | mothers | Hospital | Dhaka, Bangladesh | unknown | Unknown/mixed | Islam and Hinduism |
| Tilayie Feto Gelan, Yadeta Dessie Bacha, Nega Assef, Aboma Motumma, Aklilu Abrham Roba, Yohanes Ayele and Fikirte Tsige | Acceptability of donor breast milk banking, its use for feeding infants, and associated factors among mothers in eastern Ethiopia | 2018 | <https://doi.org/10.1186/s13006-018-0163-z> | Funded by Haramaya University Young Innovative Research Grant Funding | None declared. | focus group discussions | Thematic approach | Hiwot Fana Specialized University Hospital, Jugola Hospital, Dil-Chora Hospital, and Karamara Hospital, | 33 | acceptability of donor breast milk banking, its use for feeding infants, and associated factors among mothers | mothers | Hospital | Ethiopia | unknown | formal | Orthodox, Muslim, Prostetant, Catholic |
| Karleen D. Gribble | Peer-to-Peer Milk Donors’ and Recipients’ Experiences and Perceptions of Donor Milk Banks | 2013 | DOI: 10.1111/1552-6909.12220 | None declared. | None declared. | A descriptive survey design containing closed and open-ended questions | Content analysis (Hsieh HF, Shannon SE 2005) | Facebook pages Human Milk 4 Human babies and Eats on Feets | 139 | intersection of peer-to-peer milk sharing and donor milk banks | mothers | community | North America, Europe, Oceania, and Asia | unknown | formal | unknown |
| Karleen D. Gribble | A better alternative': Why women use peer-to-peer shared milk | 2014 |  | None declared. | None declared. | descriptive survey design containing both closed- and open-ended questions | Content analysis (Hsieh HF, Shannon SE 2005) | Facebook pages Human Milk 4 Human Babies | 33 | process by which women came to use peer-shared milk | peer-to-peer milk donors and recipients | Community | Australia, Canada, Malaysia, New Zealand and the United States | unknown | informal | unknown |
| Emine Karacan, Sirin¸ Celikkanat, Zeynep Gung | human milk banking | 2024 | https://doi.org/10.1016/j.nut.2023.112299 | None declared. | None declared. | semistructured in-depth interviews | Content analysis method (Miles MB, Huberman AM. Q 1994) | Gaziantep Health Care Family Health Center | 30 | beliefs and opinions of breastfeeding mothers about human milk banking | mothers | Community | Turkey | unknown | formal | unknown |
| Mahnoush Karami, Zeinab Zaremohzzabieh, Mansoureh Zarean  Ali-Reza Ahmadi | Exploring barriers to human milk banking acceptability among nursing mothers in Iran using social cognitive perspectives | 2025 | https://doi.org/10.1186/s13006-025-00767-9 (Karami et al. 2025, S. 1) | None declared | None declared | semi-structured, in-depth face-to-face interviews | thematic analysis approach | purposive sampling from antenatal and postnatal care clinics at two urban teaching hospitals | 12 | barriers to human milk banking acceptability | Mothers | Hospital | Iran | Unknown | Formal | unknown |
| Meltem Kürtüncü, Aylin Kurt, Nurten Arslan | The opinions of mothers with children of ages 0-2 about human milk banks: A qualitative study | 2024 | DOI: 10.33308/2687248X.202461321 (Kürtüncü et al. 2024, S. 1) | None declared | None declared | semi-structured Interview | Colaizzi's  phenomenological method | purposive sampling in the pediatrics department of a state hospital | 17 | opinions of mothers with children of ages 0-2 about human milk banks | Mothers | Hospital | Türkiye | Unknown | Formal | unknown |
| Hui Fang Loh, Jiayu Rachel Lee, Andrea Ruwen Tan, Xin Lei Goh, Yi Fen Low, Yvonne Peng Mei Ng , Shefaly Shorey | Emotional journey of Asian mothers of premature infants who received pasteurised donor human milk: a qualitative study | 2023 | doi:10.1136/archdischild-2022-324748 | None declared. | None declared. | semistructured individual interviews | Braun and Clarke’s six-step process of thematic analysis | purposive sampling | 19 | effect of donor human milk usage on the emotional experience | mothers | Hospital | Singapore | premature infant | formal | Christianity, Islam, Buddhism Hinduism, None |
| Welma Lubbe, Charlene S. Oosthuizen, Robin C. Dolman and Namukolo Covic | Stakeholder Attitudes towards Donating and Utilizing Donated Human Breastmilk | 2019 | doi:10.3390/ijerph16101838 | Funded by Faculty of Health Sciences, North-West University and supported by South Africa’s National Research Foundation | None declared. | focus group discussions | Qualitative content analysis approach | selected clinics | 41 | attitudes regarding the donation and receipt of human breastmilk | mothers, grandmothers, healthcare professionals | hospital | North West Province of South Africa | infants aged 0–12 months | formal | unknown |
| Catherine Mackenzie, Sara Javanparast and Lareen Newman | Mothers’ Knowledge of and Attitudes toward Human Milk Banking in South Australia: A Qualitative Study | 2013 | DOI: 10.1177/0890334413481106 | Funded by a grant from the Faculty of Health Sciences, Flinders University. | None declared. | in-depth interviews | Framework analysis (Ritchie J, Spencer L 1994) | Australian Breastfeeding Association–South Australian/Northern Territory Branch (SANT ABA), the Preterm Infants Parents’ Association (PIPA), and Miracle Babies Foundation. | 59 | mothers’ knowledge of and attitudes toward human milk banks | mothers | community | South Australia | Preterm/sick infants | formal | unknown |
| Sarah Magowan, Kathy Burgoine, Collin Ogara, James Ditai and Melissa Gladstone | Exploring the barriers and facilitators to the acceptability of donor human milk in eastern Uganda – a qualitative study | 2020 | https://doi.org/10.1186/s13006-020-00272-1 | Self-funded by Magowan S. | None declared. | focus group discussions and in-depth interviews | Inductive approach (Korstjens I, Moser A. Series 2018) | current in-patients in the NNU and weekly neonatal follow up | 28 | barriers and facilitators to the acceptability of donor human milk | caregivers | Hospital | Uganda | unknown | formal | unknown |
| Tebogo Mampane, Jacqueline E. Wolvaardt | The acceptability of a donor human milk bank and donated human milk among mothers in Limpopo Province, South Africa | 2024 | DOI: 10.1111/mcn.13651 | None declared. | None declared. | in‐depth interviews | Interpretative phenomenological analysis (IPA) framework | pamphlets and word of mouth  by the researcher | 23 | mothers' acceptance of the donor  human milk bank and DHM | mothers | Hospital | South Africa | unknown | Unknown/mixed | Zion Christian Church, Apostolic Church, Christian, Not attending church, Muslim, Roman Catholic |
| Jayashree Mondkar, Ruchika Chugh Sachdeva, Sunita Shanbhag, Aisha Khan, Minu Manuhar Sinha, Rajib Dasgupta, Kiersten Israel-Ballard and Vandana Sabharwal | Understanding Barriers and Facilitators for Human Milk Banking Among Service Providers, Mothers, and Influencers of Preterm and Sick Neonates Admitted at Two Health Facilities in a Metropolitan City in India | 2018 | DOI: 10.1089/bfm.2018.0103 | None declared. | None declared. | focus group discussions | Analyzed separately for each category | Two health facilities under the Mumbai Municipal Corporation | 56 | perceptions and practices on DHM and HMBs | mothers, fathers, grandmothers | Hospital | India | unknown | Unknown/mixed | unknown |
| Elizabeth J. O’Sullivan, Sheela R. Geraghty and Kathleen M. Rasmussen | Informal Human Milk Sharing: A Qualitative Exploration of  the Attitudes and Experiences of Mothers | 2016 | DOI: 10.1177/0890334416651067 | Funded by a Fulbright International Science and Technology Award | None declared. | semistructured in-depth interview | Inductive approach (Thomas DR 2006) | notices in pediatric offices, cafés, and stores that sold infant products, via emails to parenting listservs, and by snowball sampling | 41 | experiences of and attitudes toward HM sharing | mothers | Community | New York | unknown | informal | unknown |
| Ayelet Oreg and Maya Negev | Peer-to-Peer Human Milk-Sharing Among Israeli Milk Donors: A Mixed-Methods Study in the Land of Milk and Honey | 2023 | https://doi.org/10.1177/08903344231196113 | None declared. | None declared. | semi-structured online survey, including both closed- and open-ended questions | Content analyzed (Hsieh & Shannon, 2005) | non-probability sampling to obtain a broad sample of human milk donors | 250 | peer-to-peer human milk-sharing in Israel | mothers | Community | Israel | unknown | informal | unknown |
| Hala Oueidat, Lama Charafeddin, Hana Nimer, Hiba Hussein, Mona Nabulsi | Knowledge and attitudes of Lebanese women towards Baby Friendly Hospital Initiative practices | 2020 | https://doi.org/10.1371/journal.pone.0238730 | None declared. | None declared. | cross-sectional survey | Content analysis | randomly chosen clinics in each Mouhafaza | 517 | knowledge of Lebanese women of BFHI steps, and attitudes towards Baby Friendly Hospitals, Skin-to-Skin Contact and Kangaroo Care practices. | pregnet women | Hospital | Lebanon | unborn | Unknown/mixed | unknown |
| Molly R. Rabinowitz, Laura R. Kair, Heather L. Sipsma, Carrie A. Phillipi and Ilse A. Larson | Human Donor Milk or Formula: A Qualitative Study of Maternal Perspectives on Supplementation | 2018 | DOI: 10.1089/bfm.2017.0114 | None declared. | None declared. | semistructured in-person interviews | Thematic analysis | Oregon Health & Science University | 24 | Acceptance of HDM versus infant formula | mothers | Hospital | Portland, Oregon | term newborns | Unknown/mixed | unknown |
| Linda Vesel, Emily Benott, Sarah Somji, Roopa M Bellad, Umesh Charantimath, Sangappa M Dhaded, Shivaprasad S Goudar, Chandrashekhar Karadiguddi, Geetanjali Mungarwadi, Sunil S Vernekar, Rodrick Kisenge, Karim Manji, Nahya Salim, Abraham Samma, Christopher R Sudfeld, Irving F Hoffman, Tisungane Mvalo, Melda Phiri, Friday Saidi, Jennifer Tseka, Mercy Tsidya, Bethany A Carus, Christopher P Duggan, Kiersten Israel-Ballar, Anne CC Lee, Kimberly L Mansen, Stephanie L Martin, Krysten North, Melissa F Young, Eliza Fishman, Katelyn Fleming, Katherine EA Semrau, Lauren Spigel, Danielle E Tuller and Natalie Henrich | Facilitators, barriers, and key influencers  of breastfeeding among low birthweight infants: a qualitative study in India, Malawi, and Tanzania | 2023 | https://doi.org/10.1186/s13006-023-00597-7 | Supported by the Bill & Melinda Gates Foundation | Supported by the Bill & Melinda Gates Foundation and by the National Institutes of Health | focus group discussions | Framework analysis (Gale et al. 2013) | purposive sampling, either facetoface or through email | 213 | Facilitators, barriers, and key influencers  of breastfeeding among low birthweight infants | mothers, family members, community leaders , HCPs, government officials, experts | Community | Belgaum, Karnataka State, India; Cuttack, Odisha State, India; Lilongwe, Malawi; and Dar es Salaam, Tanzania. | unknown | Unknown/mixed | unknown |
