# Supplementary material for: Parents' Transcultural Attitudes and Knowledge Concerning Human Milk Exchange: A Qualitative Systematic Review and Meta‐Aggregation
Source: Matern Child Nutr. 2026 Oct 27;22(4):e70278. doi: 10.1111/mcn.70278 (PMC13714566; doi:10.1111/mcn.70278)
Supplement: Supplementary file 5 [file MCN-22-e70278-s005.docx]

Supplementary file 1: Search strategy: revised version

|  | Pubmed | Cinahl | Medline | Embase |
| --- | --- | --- | --- | --- |
| Religion and culture | (Religion [TIAB] OR  Religious [TIAB] OR  Faith [TIAB] OR  Persuasion [TIAB] OR  Creed [TIAB] OR  Piety [TIAB] OR  devoutness [TIAB] OR  Devotion [TIAB] OR  Denomination [TIAB] OR  Belief [TIAB] OR  Cult* [TIAB] OR  Diversity [TIAB] OR  Transcultural [TIAB] OR  Ethnology [TIAB] OR  Origin [TIAB] OR  background [TIAB] OR  MUSLIM [TIAB] OR  Acculturation [MH] OR  cross-cultural comparisoN [MH] OR  cultural diversity [MH] OR  cultural evolution [MH] OR  ethnology [MH] OR  cultural characteristics [MH] OR  anthroposophy [MH] OR  buddhism [MH] OR  christianity [MH] OR  hinduism [MH] OR  islam [MH] OR  judaism [MH] OR  religion and medicine [MH] OR  religion and sex [MH] OR  religious missions [MH] OR  religious philosophies [MH] OR  theology [MH] OR  saints [MH] OR  diversity, equity, inclusion [MH] OR  transcultural Nursing [MH]) | (TI Religion or  AB Religion or  TI Religious or  AB Religious or  TI Faith or  AB Faith or  TI Persuasion or  AB Persuasion or  TI Creed or  AB creed or  TI piety or  AB Piety or  TI devoutness or  AB devoutness or  TI devotion or  AB Devotion or  TI Denomination or  AB denomination or  TI belief or  AB Belief or  TI cult* or  AB Cult* or  TI Diversity or  AB diversity or  TI transcultural or  AB Transcultural or  TI Ethnology or  AB ethnology or  TI origin or  AB Origin or  TI background or  AB background or  Ti muslim OR  Ab Muslim Or  MH religion and religions+ OR  MH religion and medicine+ OR  MH judaism+ OR  MH buddhism+ OR  MH christianty+ OR  MH cultus+ OR  MH hinduism+ OR  MH islam+ OR  MH acculturation+ OR  MH cultural diversity+ OR  MH diversity, equity, inclusion+ OR  MH cultural values+ OR  MH ethic groups+ OR  MH ethnology+ OR  MH ethology+ OR  MH cults+ OR  MH transcultural nursing society+ OR  MH adoption, transracial+ OR  MH transcultural nurses+ OR  MH transcultural nursing+ OR  MH transcultural adaption+ OR  MH ethnolocial research+) | (Religion.ti,ab,KW OR  Religious.ti,ab,KW OR  Faith.ti,ab,KW OR  Persuasion.ti,ab,KW OR  Creed.ti,ab,KW OR  Piety.ti,ab,KW OR  devoutness.ti,ab,KW OR  Devotion.ti,ab,KW OR  Denomination.ti,ab,KW OR  Belief.ti,ab,KW OR  Cult*.ti,ab,KW OR  Diversity.ti,ab,KW OR  Transcultural.ti,ab,KW OR  Ethnology.ti,ab,KW OR  Origin.ti,ab,KW OR  background.ti,ab,KW OR  muslim.ti,ab,KW OR  EXP religion/ OR  EXP Freedom of religion/ OR  EXP religion and medicine/ OR  EXP religion and science/ OR  EXP religion and sex/ OR  EXP acculturation/ OR  EXP cross-cultural comparsion/ OR  EXP cultural characteristics/ OR  EXP cultural diversity/ OR  EXP ethnology/ OR  culture/ OR  EXP anthropology, cultural / OR  EXP cultural competency /OR  EXP faith-Based organizations/ OR  EXP diversity, equity, inclusion/ OR  EXP transcultural nursing/ OR  EXP ISLAM/ OR  EXP HINDUISM/ OR  EXP JUDAISM/ OR EXP CHRISTIANITY/ OR  EXP BUDDHISM/ OR  EXP THEOLOGY/) | (Religion.ti,ab,KW OR  Religious.ti,ab,KW OR  Faith.ti,ab,KW OR  Persuasion.ti,ab,KW OR  Creed.ti,ab,KW OR  Piety.ti,ab,KW OR  devoutness.ti,ab,KW OR  Devotion.ti,ab,KW OR  Denomination.ti,ab,KW OR  Belief.ti,ab,KW OR  Cult*.ti,ab,KW OR  Diversity.ti,ab,KW OR  Transcultural.ti,ab,KW OR  Ethnology.ti,ab,KW OR  Origin.ti,ab,KW OR  background.ti,ab,KW OR  muslim.ti,ab,KW OR  EXP religion/ Or  EXP religious group/ Or  EXP cultural anthropology/ Or  EXP cultural background/ Or  EXP cultural competence/ Or  EXP cultural diversity/ Or  EXP cultural factor/ Or  EXP cultural nursing/ Or  EXP cultural Sensitivity/ OR  EXP cultUral value/ Or  EXP faith-based organization/ Or  EXP diversity, equity and inclusion/ Or  EXP racial diversity/ Or  EXP human diversity/ Or  EXP transcultural care/ Or  EXP ethnology/ Or  EXP ethic background/ Or  EXP racial background/ OR  EXP islam/ OR  EXP islamic medicine/ OR  EXP buddhism/ OR  EXP buDDhist/ OR  EXP christianity/ OR  EXP christian/ OR  EXP christian science/ OR  EXP judaism/ OR  EXP jew/ OR  EXP hinduism/ OR  EXP hindu/ OR  EXP theology/) |
|  | AND | AND | AND | AND |
| Donor human milk | (“milk bank*” [TIAB] OR  “donor milk” [TIAB] OR  “donated milk” [TIAB] OR  “milk donation*” [TIAB] OR  “donor human milk” [TIAB] OR “donor breast milk” [TIAB] OR  milk banks [MH]) | (TI “milk bank*” or  AB “milk bank*” or  TI “donor milk” or  AB “donor milk” or  TI “donated milk” or  AB “donated milk” or  TI “milk donation*” or  AB “milk donation*” or  TI “donor human milk” or  AB “donor human milk” or  TI “donor breast milk” or  AB “donor breast milk” or  MH milk banks+ or  MH donor milk+) | (“milk bank*”.ti,ab,kw OR  “donor milk”.ti,ab,kw OR  “donated milk”.ti,ab,kw OR  “milk donation*”.ti,ab,kw OR  “donor human milk”.ti,ab,kw OR  “donor breast milk”.ti,ab,kw OR  EXP milk banks/) | (“milk bank*”.ti,ab,KW OR  “donor milk”.ti,ab,KW OR  “donated milk”.ti,ab,KW OR  “milk donation”.ti,ab,KW OR  “donor human milk”.ti,ab,KW OR  “donor breast milk”.ti,ab,KW OR  EXP milk bank/ Or  EXP donor milk/) |
|  | AND | AND | AND | AND |
| Attitude and knowledge | (attitude* [TIAB] OR  opinion* [TIAB] OR  compliance [TIAB] OR  acceptation [TIAB] OR  interest [TIAB] OR  perspective* [TIAB] OR  standpoint* [TIAB] OR  “point of view” [TIAB] OR  perception* [TIAB] OR  conviction [TIAB] OR  acceptance [TIAB] OR  view* [TIAB] OR  COncern* [TIAB] OR  DeterminAnt* [TIAB] OR  barrier* [TIAB] OR  facilitatoR* [TIAB] OR  knowledge [TIAB] OR  expertise [TIAB] OR  proficiency [TIAB] OR    attitude [MH] OR  patient acceptance of Health care [MH] OR  knowledge [MH] OR  social facilitation [MH] OR  patient compliance [MH] OR  health knowledge, attitudes, practice [MH]) | (TI compliance or  AB compliance or  TI acceptation or  AB acceptation or  TI interest or  AB interest or  TI perspective* or  AB perspective* or  TI standpoint* or  AB Standpoint* or  TI “point of view” or  AB “point of view” or  TI perception* or  AB perception* or  TI conviction or  AB conviction or  TI expertise or  AB expertise or  TI proficiency or  AB proficiency or  TI attitude or  AB Attitude or  TI acceptance or  AB acceptance or  TI view* or  AB View* or  TI Knowledge or  AB knowledge or  TI Concern* or  AB COncern” or  TI DeterminAnt” or  AB Determinant* or  TI opinion or  AB OPinion or  TI barrier” or  AB Barrier* or  TI Facilitation* or  AB facilitation* or    MH attitude+ or  MH acceptance+ and  MH commitment therapy+ OR  MH knowledge+ or  MH social determinants of health+ OR  MH patient compliance+ or  MH perception+ or  MH health knowledge+) | (attitude*.ti,ab,KW OR  opinion*.ti,ab,KW OR  compliance.ti,ab,KW OR  acceptation.ti,ab,KW OR  interest.ti,ab,KW OR  perspective*.ti,ab,KW OR  standpoint*.ti,ab,KW OR  “point of view”.ti,ab,KW OR  perception*.ti,ab,KW OR  conviction.ti,ab,KW OR  acceptance.ti,ab,KW OR  view*.ti,ab,KW OR  COncern*.ti,ab,KW OR  DeterminAnt*.ti,ab,KW OR  barrier*.ti,ab,KW OR  facilitatoR*.ti,ab,KW OR  knowledge.ti,ab,KW OR  expertise.ti,ab,KW OR  proficiency.ti,ab,KW OR    EXP attitude/ OR  EXP patient acceptance of health care/ OR  EXP health knowledge, attitudes, practice/ OR  EXP social facilitation/ OR  EXP patient COMPLIANCE/ OR  EXP compliance/ OR  EXP treatment adherence and compliance/ OR  EXP perception/ OR  EXP expression of concern/ OR  EXP social determinants of health/ OR  EXP KNOWLEDGE/) | (attitude*.ti,ab,KW OR  opinion*.ti,ab,KW OR  compliance.ti,ab,KW OR  acceptation.ti,ab,KW OR  interest.ti,ab,KW OR  perspective*.ti,ab,KW OR  standpoint*.ti,ab,KW OR  “point of view”.ti,ab,KW OR  perception*.ti,ab,KW OR  conviction.ti,ab,KW OR  acceptance.ti,ab,KW OR  view*.ti,ab,KW OR  COncern*.ti,ab,KW OR  DeterminAnt*.ti,ab,KW OR  barrier*.ti,ab,KW OR  facilitatoR*.ti,ab,KW OR  knowledge.ti,ab,KW OR  expertise.ti,ab,KW OR  proficiency.ti,ab,KW OR  EXP parental attitude/ OR  EXP compliance/ OR  EXP variant of interest/ OR  EXP perception/ OR  EXP acceptancE and commitment therapy/ OR  EXP social acceptance/ OR  EXP variant of concern/ OR  EXP social determinants of health/ OR  EXP knowledge/ OR  EXP KNOWlEDGE gap/) |
